# Recent advances in tumor immunotherapy based on NK cells

**DOI:** 10.3389/fimmu.2025.1595533

**Published:** 2025-08-07

**Authors:** Mengmeng Chen, Bing Zhang, Xuanlin Mu, Bingqiang Zhang, Tielin Yang, Gaofeng Zhang, Yuchao Gu, Bin Pei, Shaoshuai Liang

**Affiliations:** ^1^ Research Institute, Qingdao Restore Biotechnology Co., Ltd., Qingdao, Shandong, China; ^2^ Research and Development Department (R&D), Key Laboratory of Cancer and Immune Cells of Qingdao, Qingdao, Shandong, China; ^3^ Department of Urology, Qilu Hospital, Shandong University, Qingdao, Shandong, China; ^4^ Department of Pharmacy, Qingdao Cardiovascular Hospital, Qingdao, Shandong, China; ^5^ Key Laboratory of Biomedical Information Engineering of Ministry of Education, Biomedical Informatics & Genomics Center, School of Life Science and Technology, Xi’an Jiaotong University, Xi’an, Shaanxi, China; ^6^ Department of Anesthesiology, Qingdao Municipal Hospital, Qingdao, Shandong, China; ^7^ Qingdao Shark Variable Domain of Immunoglobulin New Antigen Receptors (VNARs) Development Technology Innovation Center, College of Biological Engineering, Qingdao University of Science and Technology, Qingdao, Shandong, China; ^8^ Department of Evidence-Based Medicine Center, Xiangyang No.1 People’s Hospital, Hubei University of Medicine, Xiangyang, Hubei, China; ^9^ Department of Cell Therapy Centre, Xiangyang No.1 People’s Hospital, Hubei University of Medicine, Xiangyang, Hubei, China

**Keywords:** natural killer cells, tumor, immunotherapy, “Off-the-shelf” cell, clinical applications

## Abstract

Immunotherapy has emerged as the established fourth pillar of cancer treatment following surgery, radiotherapy, and chemotherapy, representing a cutting-edge research domain in translational medicine and clinical oncology. Natural killer (NK) cells, a type of innate cytotoxic lymphocyte, possess unique antitumor properties that are independent of major histocompatibility complex (MHC) restrictions, making them promising candidates for “off-the-shelf” therapeutic products. NK cells can eliminate tumor cells through various mechanisms. Genetic engineering of NK cells can enhance their activation signals, promote proliferation, inhibit suppressive signals, and improve tumor homing, all of which are expected to significantly boost their clinical efficacy. Compared to chimeric antigen receptor T (CAR-T) cell therapy, NK cell-based immunotherapy demonstrates superior safety and tolerability. However, the clinical application of NK cells still faces several challenges, including suboptimal expansion efficiency *in vitro*, limited persistence *in vivo*, low transduction efficiency of chimeric antigen receptor NK (CAR-NK) cells, and immunosuppressive effects of the tumor microenvironment. These issues require further investigation to achieve significant improvements. This review provides a comprehensive overview of the biological characteristics of NK cells, their antitumor mechanisms, the latest therapeutic strategies in tumor immunotherapy, and the challenges associated with NK cell-based immunotherapy, aiming to offer valuable insights for future research and clinical applications.

Natural killer (NK) cells are a critical component of innate lymphoid cells characterized by the absence of adaptive antigen receptors on their surface, yet they are capable of secreting classic cytokines such as IFN-γ. Functionally, NK cells mount an immune response against virus-infected and tumor cells (1). Since 2013, NK cells have demonstrated good safety and efficacy in the treatment of advanced leukemia (2). Subsequently, research on NK cell-based tumor immunotherapy has grown exponentially, becoming a focal point in the field of innovative immunotherapy (3–5). In recent years, advancements in cell expansion technologies, chimeric antigen receptor (CAR) development (6), CRISPR/Cas9 gene editing (7), and improved viral transduction and electroporation techniques (8) have further enhanced the clinical application of NK cells.

Tumor immunotherapy has become a critical pillar of cancer treatment. NK cells, as key effector cells of the innate immune system, can recognize and kill tumor cells without prior sensitization, exerting their effects by releasing perforin, granzyme, and secreting cytokines such as interferon-γ (IFN-γ) (9), which indicate CAR-NK cells offer significant promise for tumor immunotherapy. NK cell immunotherapy has undergone three transformative clinical phases: (1) The cytokine era (2000-2010), where IL-2-activated NK cells achieved 19-27% CR in renal cell carcinoma (RCC) trials; (2) The adoptive transfer era (2010-2020), with haploidentical NK therapy showing 45-58% CR in AML (NCT00990717) (10); and (3) The engineered NK era (2020-present), where CD19-CAR-NK trials demonstrated 73% objective response rate (ORR) with no CRS ≥ grade 3 (NCT03056339) (Marin et al., 2024b). Notably, the 2024 ELIANA trial reported 91% 12-month EFS in pediatric ALL using multiplex-edited (CD19-CAR + IL-15 + PD1-KO) NK cells–a watershed in off-the-shelf immunotherapy (11). Additionally, the combined application of NK cells with immune checkpoint inhibitors (such as anti-PD-1 antibodies) or chemotherapy drugs shows a synergistic effect in non-small cell lung cancer (NSCLC) and ovarian cancer (12). Furthermore, compared to CAR-T cells, CAR-NK cells have advantages such as not inducing cytokine release syndrome or neurotoxicity and being available from allogeneic donors, making them potential “off-the-shelf” products (13, 14). Despite the progress made in NK cell immunotherapy, their application still faces challenges, including suboptimal *in vitro* expansion, insufficient *in vivo* persistence of NK cells, low CAR-NK transduction efficiency, heterogeneity in patient responses, and inhibition by the tumor microenvironment, necessitating further research for improvement (15). This review provides a comprehensive overview of the biological characteristics of NK cells, their tumor-killing mechanisms, the latest strategies in tumor immunotherapy, and the challenges faced by NK cell-based immunotherapy, offering valuable insights for future research and clinical applications.

## Biological characteristics of NK cells

In 1976, Herberman and colleagues identified a natural cellular immune response that was independent of T cells and macrophages in a leukemia mouse model of cell-mediated immunity, which they defined as NK cells (16). NK cells originate from lymphoid progenitor cells in the bone marrow and are distributed after maturation in the bone marrow, blood, and lymphoid tissues such as the spleen, comprising about 5%-10% of peripheral blood mononuclear cells (17, 18). Functionally, NK cells resemble CD8+ T cells in their cytotoxic activity, but they lack CD3 and T cell receptors (19–21). Based on the differential expression density of the CD56 molecule on their surface, human NK cells are classified into two subsets: CD56^dim^ and CD56^bright^. The CD56^dim^ subset is primarily responsible for cytotoxic activity, exhibiting stronger killing capabilities, while the CD56^bright^ subset is more proficient in cytokine secretion, playing a key role in immune regulation (14, 22). Dogra et al. found that CD56^dim^ cells are predominant in the blood, bone marrow, spleen, and lungs, but are less prevalent in the tonsils, intestines, and lymph nodes (22, 23).

**Figure 1:**
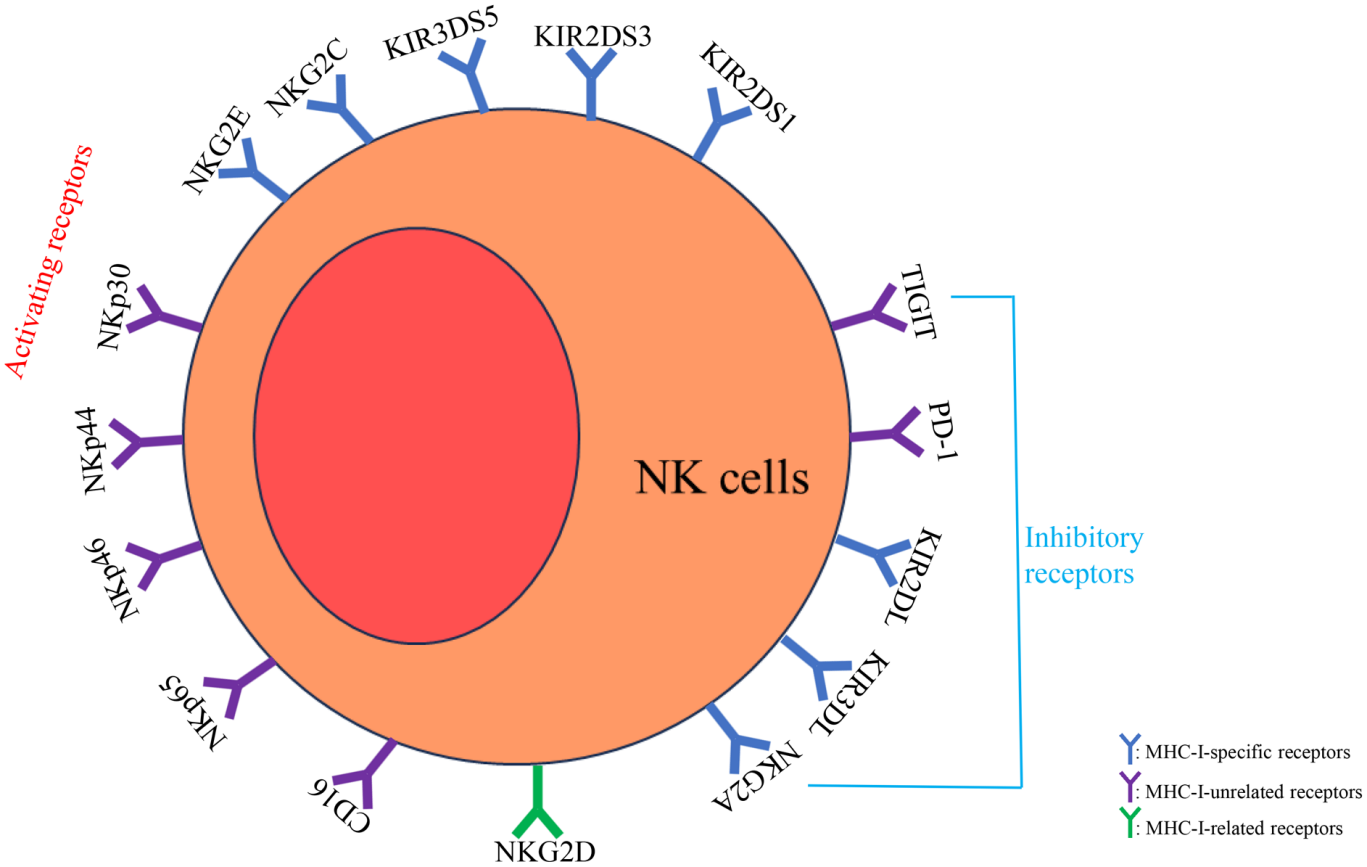
NK cells express both activating and inhibitory receptors, and their effector functions are regulated by the balance between these two types of receptors. Activating receptors can be categorized into MHC-I-specific receptors (e.g., KIR2DS1, KIR2DS3, KIR3DS5, NKG2C, NKG2E), MHC-I-related receptors (e.g., NKG2D), and MHC-I-unrelated receptors (e.g., NKp30, NKp44, NKp46, NKp65, CD16). Inhibitory receptors are mainly divided into MHC-I specific receptors (e.g., NKG2A, KIR2DL, and KIR3DL) and MHC-I-unrelated receptors (e.g., PD-1, TIGIT). The balance between activating and inhibitory receptors regulates the effector functions of NK cells.

NK cells express both activating and inhibitory receptors, and their effector functions are regulated by the balance between these two types of receptors (**Figure 1**). Activating receptors can be categorized into three groups based on their ligands: MHC-I-specific receptors (e.g., KIR2DS1, KIR2DS3, KIR3DS5, NKG2C, NKG2E), MHC-I-related receptors (e.g., NKG2D), and MHC-I-unrelated receptors (e.g., NKp30, NKp44, NKp46, NKp65, CD16) (21, 24–26). When these activating receptors bind to stress-induced ligands on target cells, they deliver activating signals, a process known as “induced self” recognition, which triggers cytotoxic activity. Inhibitory receptors, such as inhibitory killer cell immunoglobulin-like receptors (IKIRs) (like KIR2DL and KIR3DL), interact with self MHC-I molecules to achieve immune tolerance, preventing damage to self-cells. Inhibitory receptors are mainly divided into two categories based on their ligands: MHC-I specific receptors (e.g., NKG2A, KIR2DL, and KIR3DL) and MHC-I-unrelated receptors (e.g., PD-1, TIGIT). The balance between activating and inhibitory receptors regulates the effector functions of NK cells (27).

**Figure 2:**
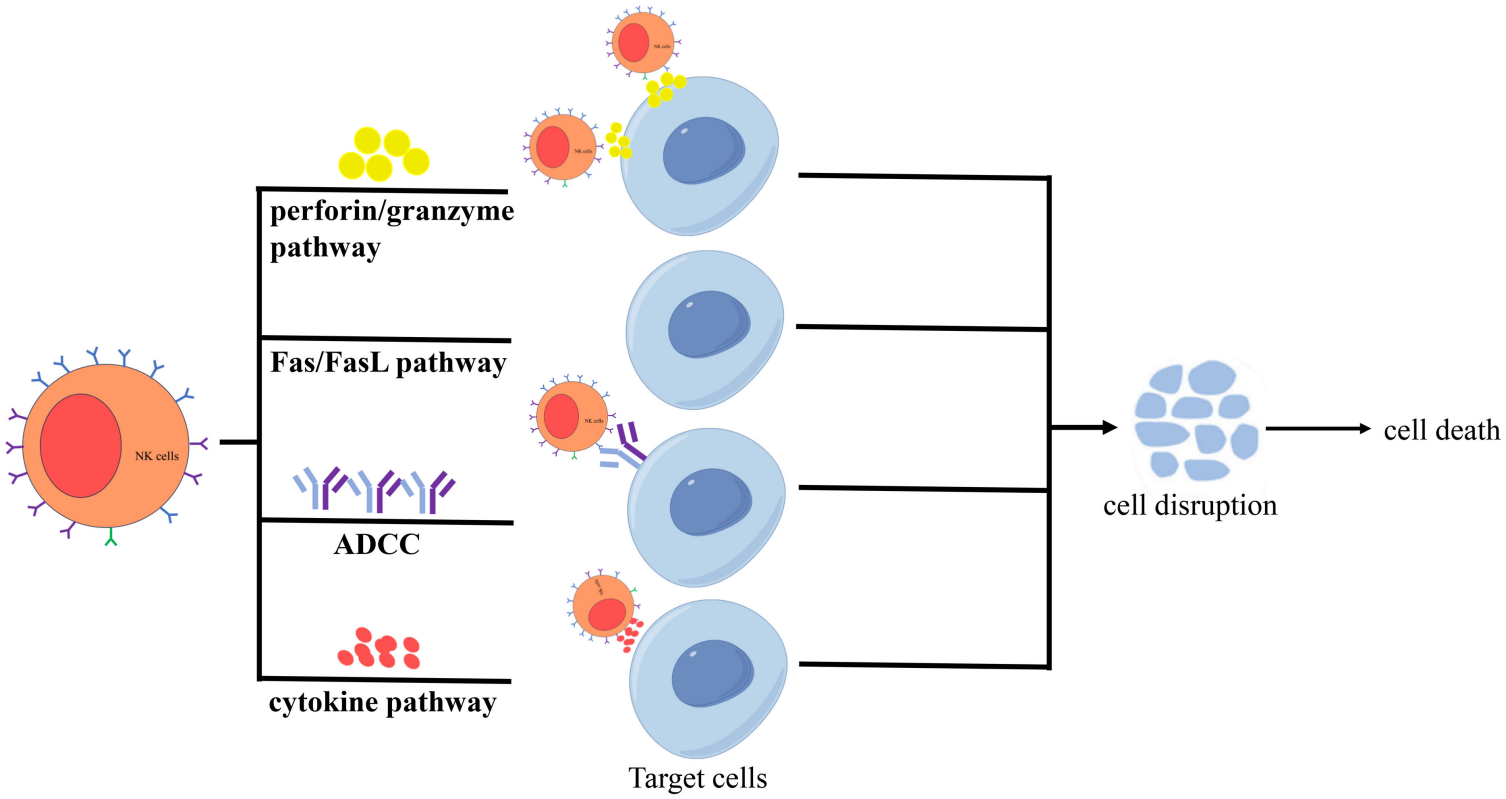
Activated NK cells exert cytotoxicity via four primary pathways, including the perforin/granzyme pathway, the Fas/FasL pathway, the ADCC pathway, and the cytokine pathway.

In addition, activated NK cells exert cytotoxicity via four primary pathways (**Figure 2**). The first is the perforin/granzyme pathway (22): Upon activation, NK cells release perforin and granzymes stored in cytoplasmic granules into the intercellular space. Perforin, structurally similar to complement components, forms transmembrane pores on target cell membranes, increasing permeability and leading to osmotic lysis. These pores also facilitate granzyme entry into the target cell, where granzymes redistribute to the cytoplasm and nucleus, accumulate at cleavage sites, and induce apoptosis. The second is the Fas/FasL pathway (28, 29): Binding of Fas ligand (FasL/CD95L, a TNF-family type II transmembrane protein) to Fas (Apo-1/CD95, a type I transmembrane receptor) triggers a “death signal” that induces target cell apoptosis within hours. The third is the antibody-dependent cell-mediated cytotoxicity (ADCC) pathway (30). NK cell-mediated ADCC can be improved by modifying antibodies, effector cells and target antigens. The fourth is the cytokine pathway (31): NK cells secrete cytokines such as TNF-α [9], which disrupt lysosomal stability in target cells, causing leakage of hydrolytic enzymes, perturbing membrane phospholipid metabolism, and activating endonucleases to degrade genomic DNA, ultimately leading to cell death.

## Mechanisms of tumor killing by NK cells

**Figure 3:**
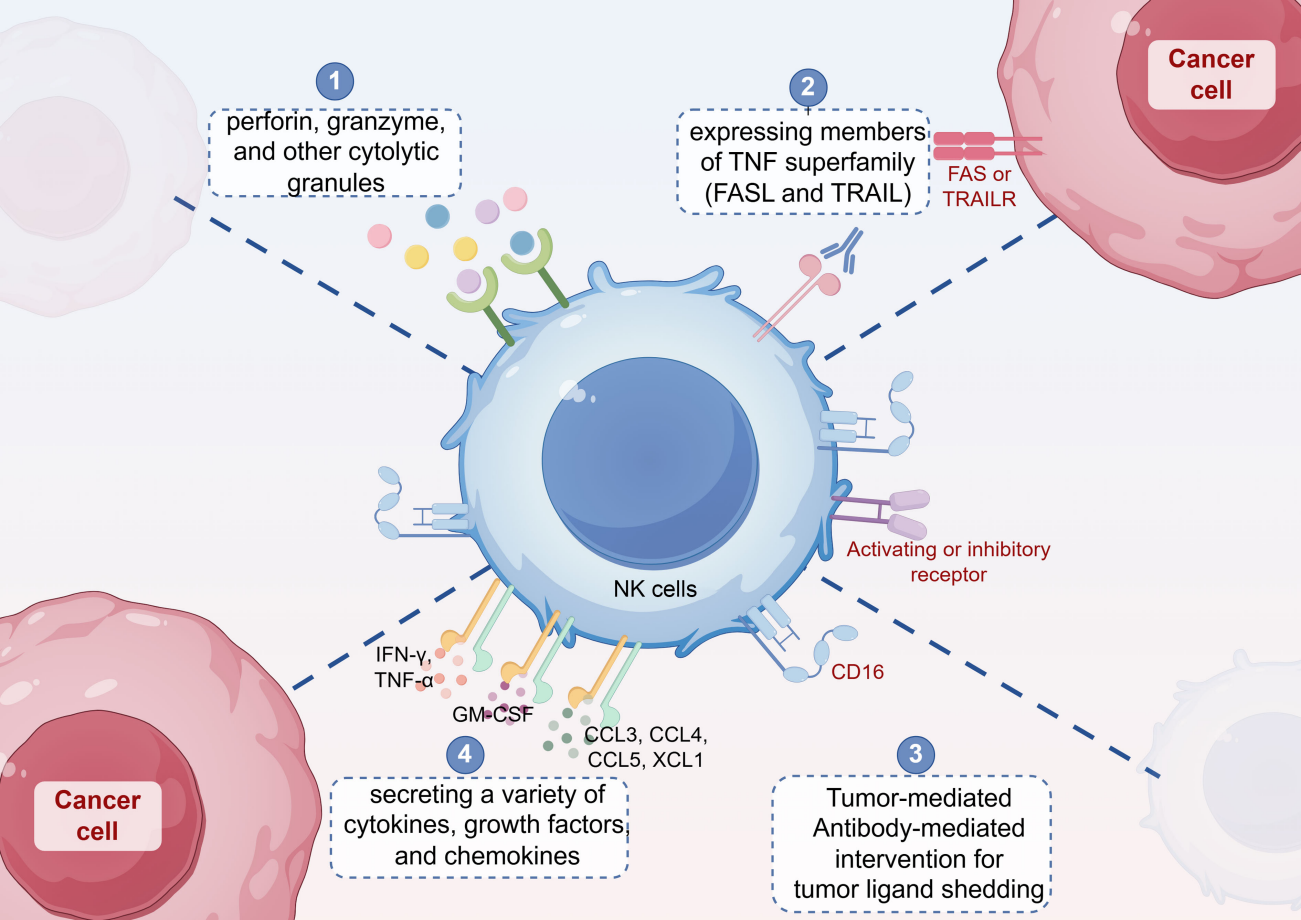
NK cells directly kill tumor cells through four main mechanisms: (1) generating large amounts of perforin, granzyme, and other cytolytic granules to induce tumor cell death; (2) expressing members of the tumor necrosis factor (TNF) superfamily, such as FASL and TRAIL, which induce tumor cell apoptosis by binding to their respective receptors, FAS or TRAILR; (3) mediating antibody-dependent cell-mediated cytotoxicity (ADCC) through FcγRIIIa (CD16); and (4) secreting a variety of cytokines (e.g., IFN-γ, TNF-α), growth factors (e.g., granulocyte-macrophage colony-stimulating factor, GM-CSF), and chemokines (e.g., CCL3, CCL4, CCL5, XCL1).

NK cells directly kill tumor cells through four main mechanisms (**Figure 3**): (1) generating large amounts of perforin, granzyme, and other cytolytic granules to induce tumor cell death; (2) expressing members of the tumor necrosis factor (TNF) superfamily, such as FASL and TRAIL, which induce tumor cell apoptosis by binding to their respective receptors, FAS or TRAILR; (3) mediating ADCC through FcγRIIIa (CD16), which can enhance NK cell cytotoxicity when used in combination with antibody drugs; and (4) secreting a variety of cytokines (e.g., IFN-γ, TNF-α), growth factors (e.g.,granulocyte-macrophage colony-stimulating factor, GM-CSF), and chemokines (e.g., CCL3, CCL4, CCL5, XCL1) (22, 32), which induce effector T cells to release more inflammatory factors, thereby inhibiting tumor cell growth or indirectly killing tumor cells by modulating the immune response (33, 34).

NK cells mediate innate immune responses by directly killing tumor cells and enhancing adaptive immune responses through signaling interactions with immune cells, such as T cells and dendritic cells (DCs), in the tumor microenvironment (TME). However, the TME contains various immune-suppressive mechanisms that significantly weaken the anti-tumor function of NK cells, limiting their efficacy.

**Figure 4:**
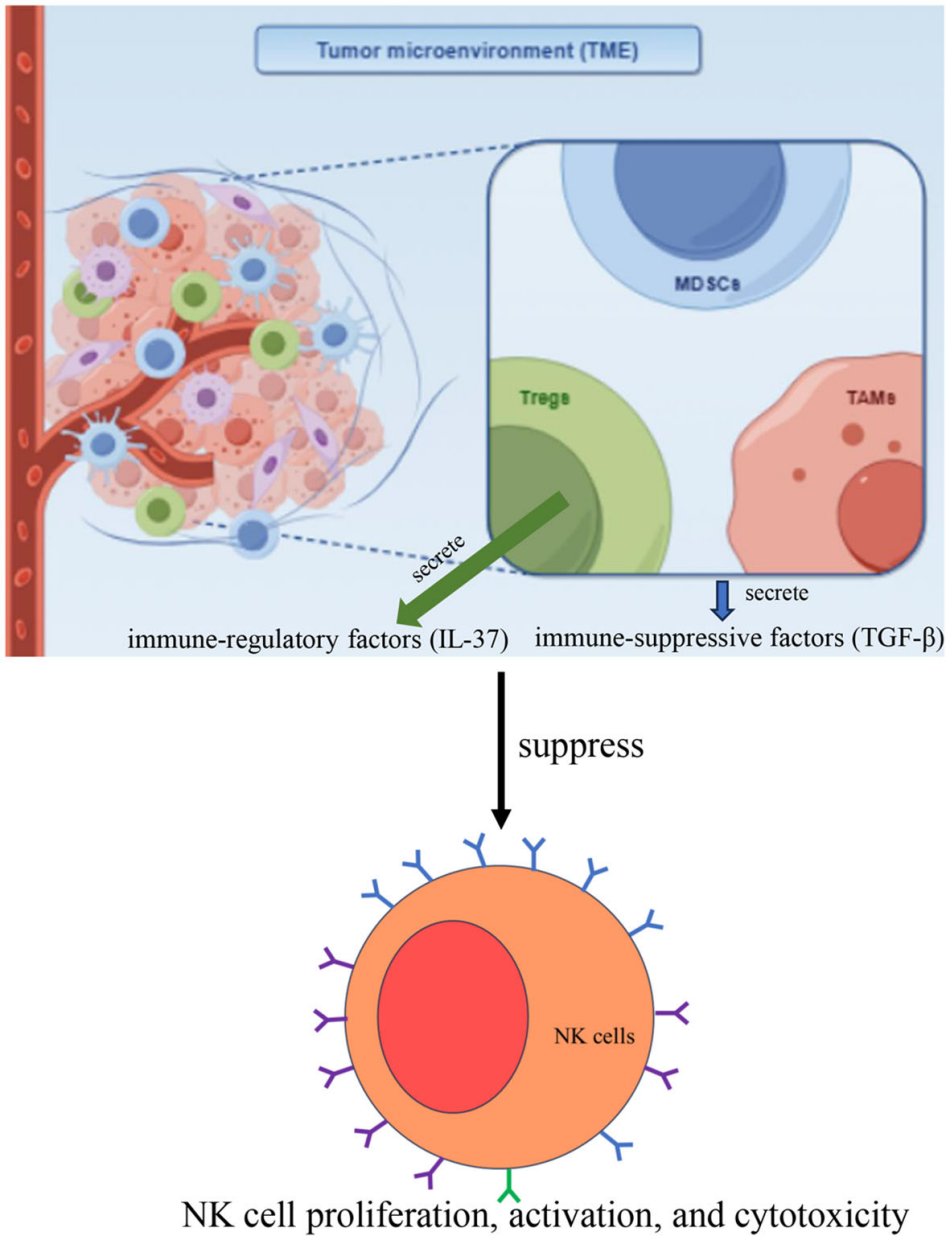
In the TME, immune-suppressive cells such as regulatory T cells (Tregs), myeloid-derived suppressor cells (MDSCs), and tumor-associated macrophages (TAMs) secrete immune-suppressive factors, particularly transforming growth factor-beta (TGF-β), which inhibits NK cell proliferation, activation, and cytotoxicity. Additionally, Tregs suppress NK cell function directly by secreting immune-regulatory factors, such as IL-37.

In the TME, immune-suppressive cells such as regulatory T cells (Tregs), myeloid-derived suppressor cells (MDSCs), and tumor-associated macrophages (TAMs) secrete immune-suppressive factors (**Figure 4**), particularly transforming growth factor-beta (TGF-β), which negatively impacts NK cell function (35). TGF-β can inhibit NK cell proliferation, activation, and cytotoxicity, exacerbating immune tolerance (36, 37). Additionally, Tregs suppress NK cell function directly by secreting immune-regulatory factors, such as IL-37 (**Figure 4**), thereby reducing NK cell efficacy in the TME and diminishing their ability to kill tumor cells (38). The immune-suppressive environment in the TME, particularly the roles of Tregs, MDSCs, and TAMs, significantly weakens NK cell anti-tumor activity through the secretion of suppressive factors such as TGF-β. Therefore, exploring strategies to alleviate these immune-suppressive mechanisms and enhance NK cell function will be critical in improving the efficacy of tumor immunotherapy.

## Comparative analysis of CAR-NK and CAR-T cells

From the aspect of cytotoxicity, compared to CAR-T cells, CAR-NK cells demonstrate a superior safety profile with markedly reduced risks of cytokine release syndrome (CRS) and neurotoxicity (39, 40). While CRS occurs in ~50-90% of CD19-targeted CAR-T therapies (grade ≥3 in 10-20%) (41), clinical trials of CAR-NK cells report only mild CRS (grade 1-2) even at high doses (42, 43). This difference may stem from NK cells’ innate ability to secrete IL-15 and IFN-γ rather than IL-6-dominated cytokine storms. Additionally, allogeneic NK cells show no graft-versus-host disease (GVHD) incidence, whereas allogeneic CAR-T requires extensive genetic modification to avoid GVHD. In regarding to persistence in the body, the persistence of CAR-NK cells *in vivo* typically ranges from weeks to months, shorter than memory-enabled CAR-T cells that may persist for years (44). However, this transient existence could be advantageous for mitigating long-term off-target effects. Recent strategies like IL-15/21 armoring or CRISPR-mediated knockout of CISH have extended CAR-NK persistence to >6 months in preclinical models (45, 46), narrowing the gap with CAR-T while maintaining safety. In addition, the comparative cost-effectiveness of CAR-T and CAR-NK was shown in **Table 1**. From a manufacturing perspective, CAR-NK cells offer significant economic advantages: (1) NK cells can be derived from universal donor cord blood or iPSCs, reducing individualized production costs by 50%–70% compared to autologous CAR-T (47); (2) cryopreserved NK products maintain efficacy after thawing, enabling off-the-shelf use versus the 2–4 week wait for CAR-T customization. Cost-effectiveness analyses estimated CAR-NK therapy at $120000−180000 per dose versus $375000−475000 for commercial CAR-T products (48, 49). While CAR-NK cells address key limitations of CAR-T therapies in toxicity and cost, their shorter persistence and lower transduction efficiency (~30-50% vs. >90% in CAR-T) remain challenges. Hybrid approaches, such as NKG2D-based CAR-T/NK co-therapy (50), may synergize the strengths of both platforms.

**Table 1:** The comparative cost-effectiveness of CAR-T and CAR-NK.

Dimension	CAR-T cells	CAR-NK cells
Production Process	Requires autologous T-cell isolation and viral vector transduction, with a 2–4-week cycle and a cost of approximately $400,000–600,000.	Can use healthy donor or iPSC-derived NK cells; universal products can be mass-produced, reducing costs by 50%–70%.
Hospitalization Management	Requires close monitoring of CRS, with ICU costs increasing total treatment costs by ~20%.	Mild toxicity allows outpatient infusion, significantly reducing management costs.
Retreatment Needs	Effective with single infusion, but re-preparation is required for recurrence.	May require multiple infusions (e.g., once every 2 weeks); long-term costs need to be evaluated alongside efficacy.

From a manufacturing perspective, CAR-NK cells offer significant economic advantages: (1) NK cells can be derived from universal donor cord blood or iPSCs, reducing individualized production costs by 50%–70% compared to autologous CAR-T; (2) cryopreserved NK products maintain efficacy after thawing, enabling off-the-shelf use versus the 2–4 week wait for CAR-T customization. Cost-effectiveness analyses estimated CAR-NK therapy at 120000−180000 per dose versus 375000−475000 for commercial CAR-T products.

CRS: cytokine release syndrome; ICU: intensive care unit.

## Strategies of NK cell-based tumor immunotherapy

**Figure 5:**
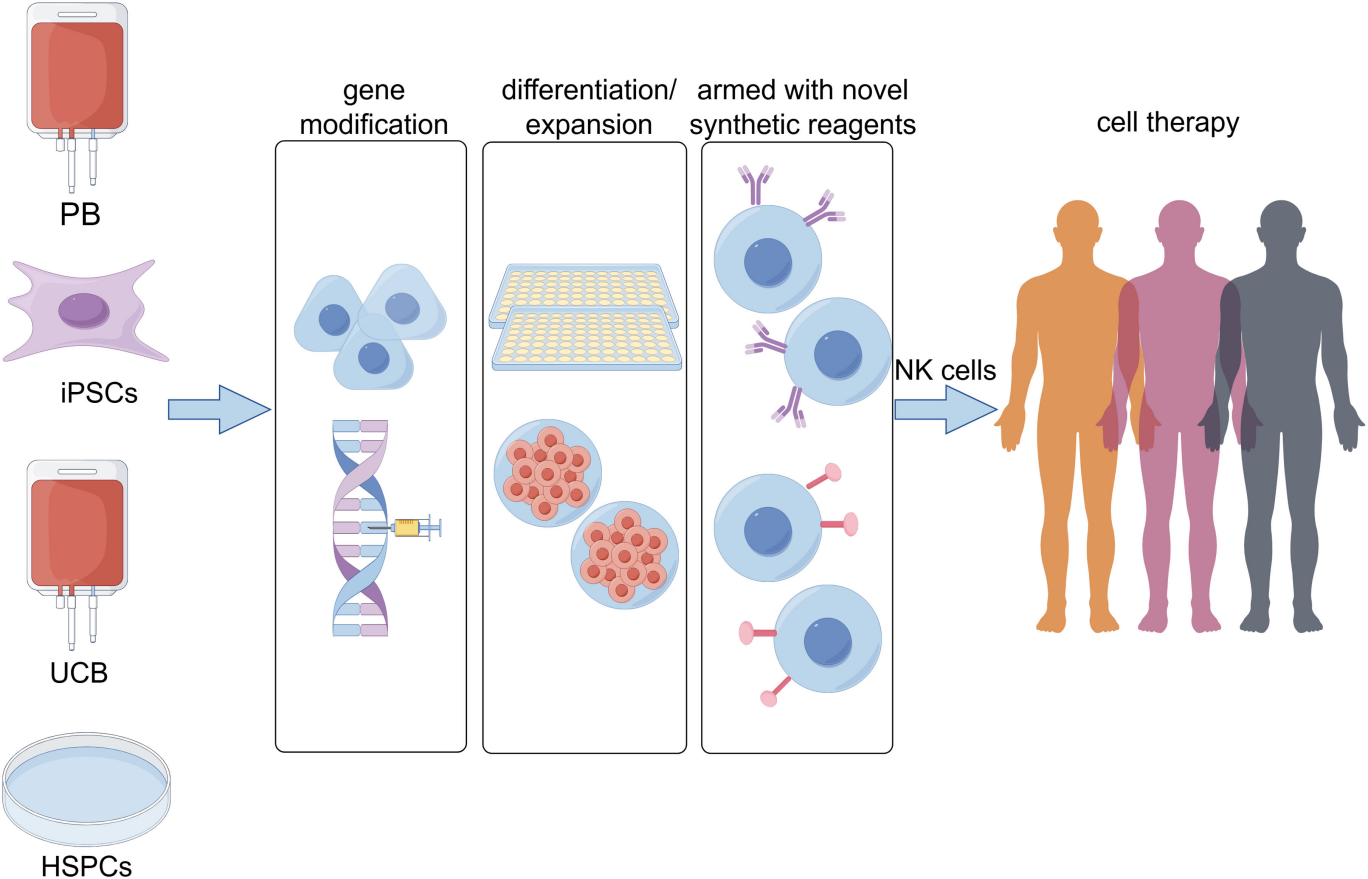
The sources of natural killer (NK) cells for immunotherapy and strategies to enhance their functionality have garnered increasing attention. Currently, multiple cell types serve as starting materials for generating NK cells, including peripheral blood (PB), induced pluripotent stem cells (iPSCs), umbilical cord blood (UCB), and hematopoietic stem and progenitor cells (HSPCs). Concurrently, the development of gene modification technologies, optimized differentiation/expansion protocols, and novel synthetic reagents has provided diverse approaches to enhance the antitumor functions of NK cells.

NK cells are a critical component of the innate immune system, playing a pivotal role in tumor immunity. In recent years, with the continuous advancement of NK cell research, numerous innovative therapeutic strategies have been developed. These strategies primarily include the use of unmodified NK cells, genetically modified NK cells, and combination therapies (**Figure 5**).

## Application of unmodified NK cells in cancer therapy

NK cells derived from peripheral blood, umbilical cord blood, placenta, induced pluripotent stem cells, and hematopoietic stem and progenitor cells (HSPCs) (**Figure 5**) can be expanded and functionally optimized through two main approaches: co-culturing with feeder layers and inducing culture with specific cytokines such as IL-2, IL-15, IL-21, IL-12, and IL-18. These methods significantly enhance NK cell expansion and maintain or improve their biological activity (51–58).

Co-culture has emerged as an important method for NK cell expansion and has demonstrated its clinical potential in various cancer therapies. For instance, NK cells derived from HSPCs (HSPC-NK) have shown certain therapeutic efficacy in early-phase clinical trials (EudraCT 2010-018988-41). In a study targeting elderly patients with acute myeloid leukemia (AML), two out of four patients with minimal residual disease (MRD) in their bone marrow converted to MRD-negative (<0.1%) after HSPC-NK cell infusion, and this response persisted for six months (59). Furthermore, Wugen’s memory-type NK cell product, WU-NK-101, optimized through their proprietary Moneta™ platform, demonstrated enhanced trafficking ability and adaptability in immunosuppressive tumor microenvironments, overcoming some of the limitations associated with NK cell therapy for solid tumors (60). These findings suggest that co-culture techniques can effectively amplify and optimize NK cell function. Additionally, a phase II clinical trial by Multhoff et al. (EudraCT 2008-002130-30) further validated the clinical value of co-culture methods (61). In this study, Hsp70-preactivated NK cells were reinfused into patients with NSCLC in combination with conventional chemotherapy and radiotherapy. The results revealed that reinfusion of Hsp70-primed NK cells significantly improved patient survival, increasing the 1-year survival rate from 33% to 67%.

The use of cytokine-mediated NK cell expansion has also made significant progress and shows promise in various cancer treatments. For example, Rafael et al. developed an IL-15 receptor agonist (NKTR-255), aimed at activating the IL-15 pathway to expand NK cells for the treatment of multiple myeloma (MM) (62). In both *in vitro* and *in vivo* studies, NKTR-255-expanded NK cells enhanced anti-tumor cytotoxicity, suppressed tumor growth, and, when combined with the anti-CD38 antibody daratumumab, effectively inhibited multiple myeloma cells. However, in clinical trials for refractory/relapsed acute myeloid leukemia (AML) (NCT03050216 and NCT01898793), Melissa et al. observed that IL-15/N-803, compared to IL-2, might reduce NK cell clinical activity due to its stimulation of CD8+ T cell activation and proliferation (63). This finding highlights the need for further mechanistic studies to optimize the cytokine selection and application in order to enhance NK cell therapeutic efficacy.

Both co-culture and cytokine-based NK cell expansion methods have demonstrated substantial potential in clinical research. However, the clinical application of adoptive NK cell immunotherapy still requires further investigation with larger cohorts to optimize treatment protocols and improve therapeutic outcomes.

## Comparative evaluation of NK cell expansion methodologies

Recent advances in NK expansion protocols highlight critical trade-offs: while feeder cells (e.g., genetically modified K562 or EBV-LCL) enable clinically relevant cell numbers, they pose theoretical risks of contaminant proliferation if irradiation fails (64). Conversely, cytokine-only methods (e.g., IL-15 + ALT-803) are more adaptable to GMP but may require longer cultures to achieve therapeutic doses. Emerging hybrid approaches, such as cytokine-loaded nanoparticles combined with transient feeder exposure (65), aim to balance yield and safety. The comparative evaluation of feeder-based versus cytokine-driven NK amplification methodologies was shown in **Table 2**.

**Table 2:** The comparative evaluation of feeder-based versus cytokine-driven NK amplification methodologies.

Parameter	Feeder-based	Cytokine-driven
Representative Protocols	K562-mb15-41BBL + IL-2/IL-15	IL-2/IL-15/IL-21 + Serum-Free Medium
Expansion Fold (14 Days)	500–1000×	100–200×
Phenotypic Characteristics	CD56bright >60%, high CD16 expression	Predominantly CD56dim, increased CD57+ with culture time
Functional Activity (*In Vitro* Cytotoxicity)	Killing rate >80% at E:T ratio 1:10 (MCF-7 model)	Killing rate ~60% at E:T ratio 1:5
GMP compliance	Complex (feeder irradiation)	Simplified
Key Challenges in Clinical Translation	Detection of residual feeder cells, removal of animal-derived components	Cost of cytokines (e.g., IL-21 ~$100/μg), potential cytokine-induced senescence

While feeder cells (e.g., genetically modified K562 or EBV-LCL) enable clinically relevant cell numbers, they pose theoretical risks of contaminant proliferation if irradiation fails. Conversely, cytokine-only methods (e.g., IL-15 + ALT-803) are more adaptable to GMP but may require longer cultures to achieve therapeutic doses.

## Application of genetically modified NK cells in cancer therapy: CAR-NK cell therapy

The approval of the first CAR-T cell therapy, Kymriah, by the FDA in 2017 marked a significant milestone in the field of cellular therapies (66). However, CAR-T treatments are associated with a range of adverse effects, such as cytokine release syndrome (CRS) and graft-versus-host disease (GVHD) (67, 68). In contrast, CAR-NK cells, due to their lack of dependence on the major histocompatibility complex (MHC), exhibit a reduced incidence of CRS, GVHD, and neurotoxicity (69–71). Furthermore, CAR-NK cells can effectively eliminate tumor cells through mechanisms independent of CAR, such as activation and inhibitory receptors, as well as CD16-mediated ADCC (72). Consequently, scientists are actively developing genetically engineered CAR-NK therapeutic strategies to enhance the tumor-killing efficacy of NK cells (73).

Chimeric antigen receptors (CARs) are synthetically engineered receptors designed to direct lymphocytes to recognize and eliminate cells expressing specific target ligands. The design of CAR-NK molecules is analogous to that of CAR-T cells and comprises four principal functional domains: the antigen-binding domain, the hinge region, the transmembrane domain, and the intracellular signaling domain (74). The antigen-binding domain typically consists of a single-chain variable fragment (scFv) derived from antibodies, which is capable of recognizing specific antigens on tumor cells. The transmembrane domain anchors the CAR molecule to the surface of effector cells. Upon recognition and activation by specific antigens, the intracellular signaling domain becomes activated, initiating downstream processes that promote the destruction of tumor cells (75–77). The intracellular signaling domain of CAR-NK cells primarily includes components such as CD3ζ, CD28, 4-1BB, OX40, 2B4, CS1, DAP10, and DAP12 (78, 79). Among them, 2B4 (CD244) and CS1 (SLAMF7) are major NK cell receptors playing a significant role in anti-tumor immunity (80, 81). Anti-SLAMF7 mAb (Elotuzumab) has been a game changer in immunotherapy against relapsed and refractory multiple myeloma (82–84). Furthermore, CAR-NK structures have evolved through four generations. The first generation primarily features the scFv antigen-binding domain and the CD3ζ intracellular signaling domain. The second and third generations incorporate one and two co-stimulatory signals, respectively. The fourth generation enhances the antitumor activity of NK cells against lymphoma xenografts by targeting cytokine-induced SH2-containing protein (CIS) (85, 86).

Currently, multiple clinical trials involving CAR-NK therapy are being conducted worldwide. As of March 10, 2025, a total of 81 trials have been registered on www.clinicaltrials.gov. Among these, 58 trials are focused on the treatment of hematologic malignancies (**Supplementary Table 1**), while 23 trials are dedicated to the treatment of solid tumors (**Table 3**).

**Table 3:** Clinical Studies of CAR-NK Cell Therapy for Solid Tumors which have been registered on www.clinicaltrials.gov.

No.	NCT Number	Study title	Study Status	Phases	Conditions	Interventions	City, Country	Year	Web link
1	NCT06816823	CAR-NK Cells (CL-NK-001) in Pancreatic Cancer	Not Yet Recruiting	Phase1	Pancreatic Cancer	Biological: CL-NK-001	Shanghai, China	2025	https://www.clinicaltrials.gov/study/NCT06816823?cond=NCT06816823&rank=1
2	NCT05776355	NKG2D CAR-NK & Ovarian Cancer	Unknown	NA	Ovarian Cancer	Biological: NKG2D CAR-NK	Hangzhou, China	2023	https://www.clinicaltrials.gov/study/NCT05776355?cond=NCT05776355&rank=1
3	NCT06454890	Clinical Study of Trop2 CAR-NK in the Treatment of Relapsed/Refractory Non-Small Cell Lung Cancer (NSCLC)	Not Yet Recruiting	Phase1 Phase2	Non-Small Cell Lung Cancer NSCLC	Biological: Anti-Trop2 CAR-NK cell	Henan, China	2024	https://www.clinicaltrials.gov/study/NCT06454890?cond=NCT06454890&rank=1
4	NCT03940820	Clinical Research of ROBO1 Specific CAR-NK Cells on Patients with Solid Tumors	Unknown	Phase1 Phase2	Solid Tumor	Biological: ROBO1 CAR-NK cells	Suzhou, China	2019	https://www.clinicaltrials.gov/study/NCT03940820?cond=NCT03940820&rank=1
5	NCT05410717	CLDN6/GPC3/Mesothelin/AXL-CAR-NK Cell Therapy for Advanced Solid Tumors	Recruiting	Phase1	Stage IV Ovarian Cancer Testis Cancer, Refractory Endometrial Cancer Recurrent CAR NK	Biological: Claudin6, GPC3, Mesothelin, or AXL targeting CAR-NK cells	Guangzhou, China	2022	https://www.clinicaltrials.gov/study/NCT05410717?cond=NCT05410717&rank=1
6	NCT06572956	Clinical Study on the Safety and Efficacy of CAR-T/CAR-NK Cells in the Treatment of Recurrent Refractory or Unresectable Solid Tumors	Active Not Recruiting	Early Phase1	Safety and Efficacy of Cellular Drugs, Objective Response Rate of Subjects, etc.	Biological: CAR-T/CAR-NK cell injection	Jinan, China	2024	https://www.clinicaltrials.gov/study/NCT06572956?cond=NCT06572956&rank=1
7	NCT05194709	Study of Anti-5T4 CAR-NK Cell Therapy in Advanced Solid Tumors	Unknown	Early Phase1	Advanced Solid Tumors	Biological: Anti-CAR-NK Cells	Wuxi, China	2021	https://www.clinicaltrials.gov/study/NCT05194709?cond=NCT05194709&rank=1
8	NCT03415100	Pilot Study of NKG2D-Ligand Targeted CAR-NK Cells in Patients With Metastatic Solid Tumors	Unknown	Phase1	Solid Tumors	Biological: CAR-NK cells targeting NKG2D ligands	Guangzhou, China	2018	https://www.clinicaltrials.gov/study/NCT03415100?cond=NCT03415100&rank=1
9	NCT06856278	Clinical Study of NKG2D CAR-NK Combined with PD-1 Monoclonal Antibody in the Treatment of ATC	Not Yet Recruiting	Phase1 Phase2	Anaplastic Thyroid Carcinoma	Drug: NKG2D CAR-NK with PD-1 Antibody	Hangzhou, China	2025	https://www.clinicaltrials.gov/study/NCT06856278?cond=NCT06856278&rank=1
10	NCT06464965	Clinical Study of Cord Blood-Derived CAR-NK Cells in Gastric Cancer and Pancreatic Cancer	Recruiting	Phase1	Gastric Cancer Pancreas Adenocarcinoma	Biological: CB CAR-NK182	Hangzhou, China	2024	https://www.clinicaltrials.gov/study/NCT06464965?cond=NCT06464965&rank=1
11	NCT06066424	Phase 1 Dose Escalation and Expansion Study of TROP2 CAR Engineered IL15-transduced Cord Blood-derived NK Cells in Patients With Advanced Solid Tumors (TROPIKANA)	Recruiting	Phase1	Solid Tumors	Drug: Rimiducid Drug: TROP2-CAR-NK Cells Drug: Fludarabine phosphate Drug: Cyclophosphamide	Houston, USA	2023	https://www.clinicaltrials.gov/study/NCT06066424?cond=NCT06066424&rank=1
12	NCT03692663	Study of Anti-PSMA CAR NK Cell (TABP EIC) in Metastatic Castration-Resistant Prostate Cancer	Unknown	Early Phase1	Metastatic Castration-resistant Prostate Cancer	Drug: TABP EIC Biological: Cyclophosphamide Biological: fludarabine	Tianjin, China	2018	https://www.clinicaltrials.gov/study/NCT03692663?cond=NCT03692663&rank=1
13	NCT06503497	A Trail of Second-line Chemotherapy Sequential NKG2D CAR-NK Cell Therapy for Pancreatic Cancer	Recruiting	Early Phase1	Pancreatic Cancer Non-resectable	Biological: chemotherapy sequential CAR-NK cell infusion	Hangzhou, China	2024	https://www.clinicaltrials.gov/study/NCT06503497?cond=NCT06503497&rank=1
14	NCT05507593	Study of DLL3-CAR-NK Cells in the Treatment of Extensive Stage Small Cell Lung Cancer	Unknown	Phase1	SCLC, Extensive Stage	Biological: DLL3-CAR-NK cells	Tianjin, China	2022	https://www.clinicaltrials.gov/study/NCT05507593?cond=NCT05507593&rank=1
15	NCT06478459	Endoscopic Ultrasound (EUS) Intratumoral Injection of CAR-NK Cells in the Treatment of Advanced Pancreatic Cancer	Recruiting	Early Phase1	Pancreatic Cancer Non-resectable	Biological: CAR-NK	Hangzhou, China	2024	https://www.clinicaltrials.gov/study/NCT06478459?cond=NCT06478459&rank=1
16	NCT04847466	Immunotherapy Combination: Irradiated PD-L1 CAR-NK Cells Plus Pembrolizumab Plus N-803 for Subjects With Recurrent/Metastatic Gastric or Head and Neck Cancer	Recruiting	Phase2	Gastroesophageal Junction (GEJ) Cancers Advanced HNSCC	Drug: N-803 Drug: Pembrolizumab Biological: PD-L1 t-haNK	Bethesda, United States	2021	https://www.clinicaltrials.gov/search?cond=NCT04847466&rank=1
17	NCT05248048	NKG2D CAR-T Cells to Treat Patients With Previously Treated Liver Metastatic Colorectal Cancer	Unknown	Early Phase1	Refractory Metastatic Colorectal Cancer	Biological: CAR-T infusion	Guangzhou, China	2021	https://www.clinicaltrials.gov/study/NCT05248048?cond=NCT05248048&rank=1
18	NCT06358430	Dose Escalation and Expansion Study of TROP2 CAR Engineered IL-15- Transduced Cord Blood-derived NK Cells in Combination With Cetuximab in Patient With Colorectal Cancer (CRC) With Minimal Residual Disease (MRD)	Recruiting	Phase1	Colorectal Cancer Minimal Residual Disease	Drug: Fludarabine Phosphate Drug: Cyclophosphamide Drug: Cetuximab Drug: TROP2-CAR-NK Cells Drug: Rimiducid (AP1903) Procedure: Lymphodepleting Chemotherapy	Houston, USA	2024	https://www.clinicaltrials.gov/study/NCT06358430?cond=NCT06358430&rank=1
19	NCT06773091	A Phase I Study of NK042 Cell Injection in Advanced Solid Tumors	Recruiting	Phase1	Advanced Solid Tumors	Bioloigcal: NK042|Drug: Fludarabine (FLU)|Drug: Cyclophosphamide (CTX)	Beijing, China	2025	https://www.clinicaltrials.gov/study/NCT06773091?cond=NCT06773091&rank=1
20	NCT05922930	Study of TROP2 CAR Engineered IL15-transduced Cord Blood-derived NK Cells Delivered Intraperitoneally for the Management of Platinum Resistant Ovarian Cancer, Mesonephric-like Adenocarcinoma, and Pancreatic Cancer	Recruiting	Phase1 Phase2	Pancreatic Cancer Ovarian Cancer Adenocarcinoma	Drug: TROP2-CAR-NK Drug: Cyclophosphamide Drug: Fludarabine	Houston, USA	2023	https://www.clinicaltrials.gov/study/NCT05922930?cond=NCT05922930&rank=1
21	NCT05703854	Study of CAR.70-engineered IL15-transduced Cord Blood-derived NK Cells in Conjunction with Lymphodepleting Chemotherapy for the Management of Advanced Renal Cell Carcinoma, Mesothelioma and Osteosarcoma	Recruiting	Phase1 Phase2	Advanced Renal Cell Carcinoma Advanced Mesothelioma Advanced Osteosarcoma	Drug: CAR.70/IL15-transduced CB-derived NK cells Drug: Fludarabine phosphate Drug: Cyclophosphamide	Houston, USA	2023	https://www.clinicaltrials.gov/study/NCT05703854?cond=NCT05703854&rank=1
22	NCT03692637	Study of Anti-Mesothelin Car NK Cells in Epithelial Ovarian Cancer	Unknown	Early Phase1	Epithelial Ovarian Cancer	Biological: anti-Mesothelin Car NK Cells	Beijing, China	2019	https://www.clinicaltrials.gov/study/NCT03692637?cond=NCT03692637&rank=1
23	NCT06652243	Clinical Study of SN301A Injection in the Treatment of Hepatocellular Carcinoma	Recruiting	Early Phase1	Hepatocellular Carcinoma (HCC)	Biological: SN301A	Shanghai, China	2024	https://www.clinicaltrials.gov/study/NCT06652243?cond=NCT06652243&rank=1

Multiple clinical trials involving CAR-NK therapy are being conducted worldwide. As of March 10, 2025, a total of 81 trials have been registered on www.clinicaltrials.gov. Among these, 23 trials are dedicated to the treatment of solid tumors.

**Supplementary Table 1** illustrates that the targets for CAR-NK therapy in hematologic malignancies are relatively concentrated. The primary targets for lymphoma and leukemia include CD19, CD123, CD22, CD33, and NKG2D ligands, while BCMA is the target for multiple myeloma. Additionally, there are studies on bispecific targets for lymphoma and leukemia, such as CD33/CLL1, CD19/CD22, and CD19/CD70 (87–97). In 2018, Chinese researchers reported the first Phase I/II clinical trial (NCT02944162) of CD33 CAR-NK cells for the treatment of relapsed/refractory acute myeloid leukemia (AML) (94). Three AML patients were enrolled in the trial, receiving infusions on days 1, 3, and 5 with doses of 3×10^8^, 6×10^8^, and 1×10^9^ cells, respectively. When the maximum dose reached 5×10^9^ cells per patient, no adverse reactions were observed, indicating good safety. However, patients experienced relapse after 4 months. In February 2020, The New England Journal of Medicine published a Phase I/II clinical trial (NCT03056339) of umbilical cord blood-derived CAR-NK cells for the treatment of B-cell lymphoma (98). This trial included 11 patients with relapsed/refractory CD19-positive non-Hodgkin lymphoma or chronic lymphocytic leukemia. Following lymphodepleting chemotherapy, patients received infusions of CD19-CAR-NK cells. The study demonstrated good safety, with no occurrences of cytokine release syndrome, neurotoxicity, or hemophagocytic lymphohistiocytosis. Furthermore, no graft-versus-host disease (GvHD) was observed, even in 2 patients with HLA mismatches. Clinical efficacy results showed that, with a median follow-up of 13.8 months (range: 2.8-20.0 months), 8 patients (73%) achieved an objective response, including 7 patients (3 with chronic lymphocytic leukemia and 4 with lymphoma) who achieved complete remission. FT596 is an iPSC-derived CAR-NK cell therapy that exerts antitumor effects through a triple mechanism: targeting CD19 with CAR, a high-affinity non-cleavable CD16 Fc receptor, and IL-15/IL-15R fusion protein. In the first-in-human Phase I clinical trial (NCT04245722) for relapsed/refractory B-cell lymphoma, 86 patients (median of 4 prior lines of therapy, 38% of whom had received CAR-T therapy) were treated with either FT596 monotherapy (Cohort A) or in combination with rituximab (Cohort B) (99). The results indicated that both regimens were well tolerated, with no maximum tolerated dose (MTD) reached. Only low-grade cytokine release syndrome was observed (Cohort A: 6% Grade 1; Cohort B: 13% Grade 1-2), and no neurotoxicity events were reported. This study validated the clinical potential of iPSC-derived “off-the-shelf” genetically modified NK cell therapy, suggesting that its standardized production could overcome the challenges of autologous CAR-T therapies in terms of heterogeneity, cost, and accessibility, thus providing a new direction for cancer immunotherapy.

As illustrated in **Table 3**, CAR-NK cell therapies for solid tumors predominantly target malignancies including colorectal cancer, breast cancer, and prostate cancer, with key molecular targets encompassing CLDN6, Anti-5T4, antimesothelin, ROBO1, PSMA, NKG2D ligands, and HER2. Despite these developments, clinical evidence supporting the therapeutic efficacy of CAR-NK cells in solid tumors remains limited. Notably, three phase I/II clinical trials (NCT03940820, NCT03941457, NCT03931720) conducted in Chinese cohorts evaluated allogeneic ROBO1-specific CAR-NK-92 cell immunotherapy for pancreatic ductal adenocarcinoma (PDAC) and ROBO1-expressing solid tumors (100–102). These investigations collectively demonstrated the feasibility of CAR-NK cell application in non-hematologic malignancies. In a separate clinical trial (NCT03415100) investigating NKG2D ligand-targeted CAR-NK therapy, three metastatic colorectal cancer patients received localized CAR-NK cell administration (103). The first two patients undergoing low-dose intraperitoneal infusion exhibited clinically significant reductions in ascites production (72% and 68% volume decrease respectively) and tumor cell density in ascitic fluid (from 1.2×10^6^/mL to 3.5×10^4^/mL). The third patient with hepatic metastases received ultrasound-guided percutaneous injection followed by intraperitoneal CAR-NK administration, achieving rapid tumor regression as evidenced by Doppler ultrasound (48% target lesion reduction within 14 days). This emerging clinical evidence underscores the potential of optimized CAR-NK cell delivery strategies and receptor engineering approaches to overcome current limitations in solid tumor immunotherapy.

## CRISPR/Cas9-based gene engineering of human NK cells, and comparison with other genome editing strategies

CRISPR/Cas9, as a precision genome-editing tool with minimal cytotoxicity and off-target effects, has emerged as a promising therapeutic strategy for complex refractory diseases. Its application in CAR-NK cell therapy demonstrates significant potential for enhancing the anti-tumor efficacy of NK cells (**Figure 5**). Notably, Velasquez et al. developed a bispecific T-cell engager (CD19-ENG)-based CAR-NK therapy capable of dual targeting CD22+ B-cell leukemia while redirecting T-cells to eliminate CD19+ malignant B-cells, effectively preventing tumor immune evasion and augmenting cytotoxic activity (104). This pioneering study represents the first demonstration of engineered CAR-NK cells with CD22 specificity combined with enhanced CD19-specific T-cell targeting in B-cell malignancies. The synergistic cytolytic targeting of malignant cells through this approach opens new avenues for gene-edited cancer immunotherapy, demonstrating substantial improvements over existing B-cell therapies and related malignancy treatments. Recent advancements in base editing technology further expand CRISPR applications. Huang Xingxu’s team successfully developed a novel universal base editor through rational integration of deaminase domains at compatible chimeric sites on nCas9 (105). Compared to conventional nCas9-terminal fused base editors, this innovative configuration maintains precise target base-editing efficiency while significantly reducing both DNA and RNA off-target effects, achieving superior specificity. Building upon these technological breakthroughs, Basecare Biotechnology Co., Ltd. has translated base/prime editing technologies into clinical development. Their peripheral blood mononuclear cell (PBMC)-derived universal off-the-shelf NK cell therapy product NK510 (Super-NK), which entered investigator-initiated trial (IIT) phase in 2022, represents one of the world’s first base-edited therapeutics to reach clinical investigation.

Furthermore, CRISPR-Cas9-mediated knockout of the inhibitory receptor NKG2A has been shown to enhance NK cell cytotoxicity against multiple myeloma by reversing the immunosuppressive signaling that normally attenuates NK cell activity (106). Another research developed two glypican-3 (GPC3)-specific CAR-NK-92 cell lines (GPC3-CAR-NK), and observed that the administration of GPC3-CAR-NK cells may represent a potential therapeutic strategy for HCC. Regional delivery or their combination with MWA (microwave ablation) could potentially enhance their therapeutic efficacy against HCC, demonstrating promising translational value (107). Collectively, the CRISPR/Cas9 system demonstrates remarkable potential in advancing NK cell immunotherapy through multiple mechanisms: (1) arming NK cells with CAR constructs; (2) enhancing NK activation pathways; (3) promoting tumor infiltration capacity; and (4) counteracting inhibitory signaling pathways.

While CRISPR-Cas9 dominates current NK cell engineering due to its simplicity and high efficiency, its sgRNA-dependent off-target effects remain a concern for clinical applications. For example, a 2023 study reported detectable off-target indels in ~15% of CRISPR-edited NK cells by whole-genome sequencing, whereas TALEN-edited cells showed no such events (108). However, TALEN’s laborious protein engineering and lower knockout rates (~40% for CD38 knockout in NK cells (109)) limit its scalability. Emerging techniques like prime editing may combine the precision of TALEN with CRISPR’s versatility, though their efficacy in NK cells awaits validation (**Table 4**).

**Table 4:** Genome editing strategies in NK cell engineering.

Parameter	CRISPR-Cas9	TALEN	Base editing
Editing Efficiency	70-90% (knockdown)	30-60% (knockdown)	50-80% (point mutations)
Off-target Rate	Moderate (sgRNA-dependent)	Low	Very low
Multiplexing Ability	High (>3 genes)	Limited (1–2 genes)	Moderate (2 genes)
Clinical Readiness	Phase I/II trials ongoing	Limited due to complexity	Preclinical
Cost (per target)	$200-500	$1,000-2,000	$500-1,000

CRISPR-Cas9 dominates current NK cell engineering due to its simplicity and high efficiency; and TALEN’s laborious protein engineering and lower knockout rates (~40% for CD38 knockout in NK cells) limit its scalability.

## Combination therapy strategies

NK cells express CD16, which mediates the ADCC pathway for tumor cell killing. Therefore, they can be combined with antibodies for the treatment of various cancers, such as non-Hodgkin lymphoma, breast cancer, colorectal cancer, and neuroblastoma. AB-101, developed by Artiva, is a cord blood-derived, allogeneic, off-the-shelf, cryopreserved, non-genetically modified NK cell product. When used in combination with the cell engager AFM13 (developed by AffiMed), it has shown promising results in the treatment of relapsed or refractory CD30-positive Hodgkin lymphoma and non-Hodgkin lymphoma. Clinical studies have demonstrated a 100% overall objective response rate and a 70.8% complete response rate in patients receiving the recommended Phase 2 dose, confirming the safety and efficacy of NK cell-based combination therapy (110). FT596, developed by Fate Therapeutics, is an off-the-shelf, allogeneic chimeric antigen receptor (CAR)-NK cell product derived from induced pluripotent stem cells (iPSCs). In humanized mouse lymphoma models, FT596, when combined with the anti-CD20 monoclonal antibody rituximab, significantly enhances tumor cell killing compared to rituximab monotherapy. A Phase I multicenter clinical trial (NCT04245722) evaluating FT596 as a monotherapy and in combination with anti-CD20 monoclonal antibody therapy reported positive interim clinical data, although the final clinical data showed suboptimal efficacy (111).

Research has demonstrated that NK cells express inhibitory receptors such as PD-1 and NKG2A, which can affect their antitumor activity. When combined with immune checkpoint inhibitors or monoclonal antibodies, the cytotoxic efficacy of NK cells can be enhanced (112). In a clinical study involving the combination of NK cells with anti-PD-1 monoclonal antibodies for the treatment of NSCLC (NCT02843204), the overall objective response rate in the combination group was 36.5%, significantly higher than the 18.5% observed in the group receiving only anti-PD-1 antibodies. Moreover, the combination therapy group exhibited a notable extension in both overall survival and progression-free survival, reaching 15.5 months and 6.5 months, respectively (113). Research targeting NKG2A have shown that the anti-NKG2A antibody Monalizumab enhances NK cell antitumor activity (114–116). A clinical trial (NCT02643550) involving Monalizumab combined with cetuximab for the treatment of recurrent or metastatic squamous cell carcinoma of the head and neck demonstrated an overall response rate of 20% (8/40) in patients previously treated with platinum-based chemotherapy and PD-1/PD-L1 antibodies (117). Among these patients, 17 (42%) experienced grade 3–4 adverse events, with only one patient (2%) experiencing Monalizumab-related grade 3–4 adverse events, such as peripheral sensory neuropathy and fatigue. No treatment-related deaths were reported, indicating a controllable safety profile. Preclinical studies have shown that the combination of Anti-PSMA CAR-NK cells with anti-PD-L1 monoclonal antibodies enhances cytotoxicity against prostate cancer cells *in vivo*.

## Challenges facing NK cell immunotherapy

Although clinical trials based on NK cells are steadily increasing, several challenges persist regarding their application. These challenges include suboptimal *in vitro* expansion efficiency, limited *in vivo* persistence, low transduction efficiency of CAR-NK cells, and the immunosuppressive effects of the tumor microenvironment.

The primary prerequisite for NK cell infusion therapy lies in achieving sufficient expansion of high-purity NK cells ex vivo. Although cytokine-based expansion methods can activate NK cells and facilitate their large-scale proliferation, low NK cell purity and inter-individual variability remain pressing issues. On the other hand, the feeder cell-based expansion method results in high NK purity, but safety concerns, such as the risk of contamination with T cells, still need to be addressed (52, 64, 78, 118). The presence of T cells in the expanded NK cell population can trigger graft-versus-host disease (GVHD) upon infusion, necessitating prior T cell depletion (119).

Once NK cells are transferred back into the human body, their persistence is limited due to the lack of essential cytokines like IL-2 and IL-15 required for their proliferation and survival. To address this issue, researchers have attempted to enhance the persistence of NK cells *in vivo* by genetically modifying them (120, 121).

Currently, the development of CAR-NK drugs has become a research hotspot, but improving the efficiency of CAR transduction into NK cells remains a critical bottleneck. Studies have shown that retroviral transduction efficiency ranges from 27% to 52%, but it may cause insertional mutations, limiting its clinical application. Although lentiviral transduction is safer than retroviral transduction, its efficiency is lower (12% to 30%). Some studies have reported that using modified baboon envelope glycoprotein (BaEV-gp) pseudotyped lentiviral vectors achieves transduction efficiency 20 times higher than vectors pseudotyped with VSV-G (122). Additionally, researchers have developed a safer method by electroporating the relevant mRNA into NK cells using clinical-grade electroporation devices (39, 88, 123, 124).

The effectiveness of NK cells in targeting and killing tumor cells is influenced not only by their intrinsic cytotoxicity but also by the TME. The tumor microenvironment itself is an inhibitory milieu for NK cell function, with altered cell metabolism contributing to increased inflammation, hypoxia, and local immune suppression. Moreover, upregulation of tumor-associated immune checkpoints can lead to NK cell inactivation and diminished cytotoxicity. Additionally, various molecules present in the tumor microenvironment can accelerate NK cell exhaustion and apoptosis (120, 125–127), particularly in solid tumors. Given the significant impairment of NK cells in tumor patients, characterized by reduced numbers and compromised function, combining NK cell infusion with conventional therapies (such as surgery, chemotherapy, or radiotherapy) offers a promising strategy to reduce tumor burden effectively (128).

Despite preclinical promise, several NK cell trials have failed to meet primary endpoints. The phase II NCT02839954 trial in DLBCL (129) was terminated due to 0% CR rate (n = 12), attributed to insufficient NK cell trafficking-a problem later addressed by CXCR4 overexpression in NCT04887012. Similarly, the myeloma trial NCT03415100 showed rapid NK cell exhaustion within 72 hours, prompting development of PD-1-deleted variants (130). These failures highlight the need for: preclinical models that better recapitulate immune evasion (e.g., humanized mice with autologous tumor stroma) (131); biomarker-driven patient stratification (e.g., NKG2D ligand expression by IHC); real-time persistence monitoring via PET imaging with 89Zr-labeled NK cells.

Further, we analyzed the clinical feasibility and cost-effectiveness of personalized versus universal donor NK therapies (**Table 5**). While personalized NK therapies theoretically eliminate allo-rejection risks, their clinical implementation faces three key hurdles: (1) frequent manufacturing failures due to patient-derived NK cell dysfunction (reported in ~40% of lymphoma cases (132)), (2) prohibitively high costs from single-patient batches, and (3) logistical delays incompatible with aggressive malignancies. In contrast, universal NK products from cord blood or iPSCs offer immediate availability and 60-70% lower costs, though they require lymphodepletion to prevent host rejection (133). Notably, the ongoing PIVOT-15 trial (NCT05410717) demonstrates comparable ORR (65% vs 68%) between personalized and universal CAR-NK for NHL, favoring universal approaches for cost-effectiveness.

**Table 5:** Head-to-head comparison of NK therapy strategies.

Parameter	Personalized (Autologous)	Universal (Allogeneic)
Production Mode	Single-patient customization (batch ≤ 1 case)	Mass production (single batch ≥ 100 cases)
Production Time	3–4 weeks	≤1 week (pre-manufactured)
Dose Consistency	Highly variable (20-60% yield)	Standardized (> 90% viability)
HLA Restrictions	Required	Not required
COGS per Dose	$200000−300000	$30000−80000
Clinical Trials	18 active (Phase I/II)	32 active (Phase II/III)
Representative Product Pipeline	Autologous CAR-NK (NCT04677796)	iPSC-NK FT596 (NCT05201760)

While personalized NK therapies theoretically eliminate allo-rejection risks, their clinical implementation faces three key hurdles. In contrast, universal NK products from cord blood or iPSCs offer immediate availability and 60-70% lower costs, though they require lymphodepletion to prevent host rejection.

COGS: cost of goods sold.

## Regulatory and translational challenges

Globally, the regulatory frameworks for NK cell therapy exhibit significant disparities. The U.S. Food and Drug Administration (FDA) classifies gene-edited NK cells as “Advanced Therapy Medicinal Products (ATMPs),” requiring Investigational New Drug (IND) applications for clinical trials and comprehensive assessments of off-target effects and long-term safety. The European Medicines Agency (EMA), by contrast, emphasizes complete traceability of cell sources, standardized manufacturing processes, and preclinical data, with stricter criteria for evaluating the immunogenicity of allogeneic cells. Gene-edited NK cell technology has sparked extensive ethical debates due to potential risks associated with human embryonic stem cells (e.g., induced pluripotent stem cells, iPSCs) or germline editing (134). Additionally, “off-the-shelf” NK cell therapies face challenges in donor rights protection, informed consent procedures, and equitable commercial distribution (14). For example, establishing ethical standards for compensating healthy donors remains a contentious issue.

In addition, the translational path for NK therapies faces three layered challenges, including Good Manufacturing Practice (GMP) requirements, safety concerns with genome editing, and cost-related limitations in resource-constrained settings. Firstly, GMP-compliant production of NK cells requires strict control of raw materials (e.g., serum-free media, viral vectors), production environments (cleanroom grades), and quality testing processes (118). For NK cells modified by gene-editing technologies like CRISPR-Cas9, regulatory agencies additionally require validation of editing efficiency, off-target sites, and stability of gene insertion. The FDA mandates full-genome sequencing data in IND applications to exclude unintended mutations with potential carcinogenic risks. Secondly, technologies such as CRISPR-Cas9 may cause chromosomal translocations, off-target mutations, or activation of proto-oncogenes (135). Allogeneic NK cell therapies may trigger host-versus-graft reaction (HAR) or GVHD, as well as CRS remains a potential risk in CAR-NK therapy, although its incidence is significantly lower than in CAR-T cell therapy. Finally, the production cost of NK cell therapy is prohibitively high, and the resource-scarce regions generally lack GMP-compliant cell production facilities, cold-chain transportation systems, and gene sequencing equipment, severely limiting the clinical application of NK cell therapy.

## Limitations and future perspectives

This review has several inherent limitations: First, although this study covers cutting-edge research from 2014 to 2025, certain emerging technologies (such as AI-optimized NK cell expansion algorithms and novel gene-editing tools) remain in the preprint stage or early laboratory validation phase and were not included in the systematic analysis, potentially leading to a delay in presenting the latest breakthroughs in the field. Second, the discussion of specific cross-disciplinary areas (such as the interaction mechanism between NK cells and tumor metabolism, and the application of nanomaterial delivery technologies in NK cell engineering) remains at the level of current status overview, lacking cross-disciplinary in-depth analysis. Third, searches only on one website (www.clinicaltrials.gov) may overlook relevant research from research centers in other countries, such as the United Kingdom, Europe, etc.; as well as and the research on animal models about NK cells also needs to be further summarized in the future. Moreover, the therapeutic efficacy analysis predominantly reflects hematologic malignancies (87% of cited trials), underscoring the need for more solid tumor data. These gaps highlight the necessity for living systematic reviews in this field.

Future progress in NK cell therapy hinges on technological innovation, standardized clinical translation, and cross-disciplinary integration. Key strategies include: developing multi-omics platforms to decode NK cell-tumor interactions; applying next-gen CRISPR tools (e.g., Cas9, base editors) for precise gene editing; implementing “universal NK cells + personalized therapy” models guided by tumor/immune profiling; establishing global multi-center trials with unified GMP and efficacy standards; and leveraging nanotechnology for targeted delivery and AI for response prediction. These efforts aim to overcome persistence, heterogeneity, and delivery challenges, advancing NK cell therapy toward broader clinical utility.

In conclusion, NK cells represent a powerful tool in cancer therapy, characterized by their innate ability to distinguish self from non-self, detect danger signals on malignant cells, and rapidly eliminate these cells. Compared to CAR-T therapy, NK cell-based immunotherapy offers significant safety advantages, positioning it as the next potential “breakthrough” in cancer immunotherapy. However, NK cell therapy also faces considerable challenges, including the safety of *in vitro* expansion techniques, limited persistence *in vivo*, and the immunosuppressive effects of the tumor microenvironment, all of which require further investigation. The continuous development of strategies such as cytokine modulation, genetic engineering, and combination therapies is expected to accelerate the clinical translation of NK cell-based treatments, ultimately improving the quality of life and survival outcomes for cancer patients. Overall, NK cell therapy holds great promise for the future.

## References

[1] Vivier E Artis D Colonna M Diefenbach A Di Santo JP Eberl G. Innate lymphoid cells: 10 years on. Cell. (2018) 174:1054–66. doi: 10.1016/j.cell.2018.07.017, PMID: 30142344

[2] Tonn T Schwabe D Klingemann HG Becker S Esser R Koehl U. Treatment of patients with advanced cancer with the natural killer cell line NK-92. Cytotherapy. (2013) 15:1563–70. doi: 10.1016/j.jcyt.2013.06.017, PMID: 24094496

[3] Terrén I Orrantia A Vitallé J Zenarruzabeitia O Borrego F. NK cell metabolism and tumor microenvironment. Front Immunol. (2019) 10:2278. doi: 10.3389/fimmu.2019.02278, PMID: 31616440 PMC6769035

[4] Guillerey C. NK cells in the tumor microenvironment. Adv Exp Med Biol. (2020) 1273:69–90. doi: 10.1007/978-3-030-49270-0_4, PMID: 33119876

[5] Shimasaki N Jain A Campana D. NK cells for cancer immunotherapy. Nat Rev Drug Discov. (2020) 19:200–18. doi: 10.1038/s41573-019-0052-1, PMID: 31907401

[6] Gong Y Klein Wolterink RGJ Wang J Bos GMJ Germeraad WTV. Chimeric antigen receptor natural killer (CAR-NK) cell design and engineering for cancer therapy. J Hematol Oncol. (2021) 14:73. doi: 10.1186/s13045-021-01083-5, PMID: 33933160 PMC8088725

[7] Doudna JA Charpentier E. Genome editing. The new frontier of genome engineering with CRISPR-Cas9. Science. (2014) 346:1258096. doi: 10.1126/science.1258096, PMID: 25430774

[8] Carlsten M Childs RW. Genetic manipulation of NK cells for cancer immunotherapy: techniques and clinical implications. Front Immunol. (2015) 6:266. doi: 10.3389/fimmu.2015.00266, PMID: 26113846 PMC4462109

[9] Huang S Xing F Dai Y Zhang Z Zhou G Yang S. Navigating chimeric antigen receptor-engineered natural killer cells as drug carriers via three-dimensional mapping of the tumor microenvironment. J Control Release. (2023) 362:524–35. doi: 10.1016/j.jconrel.2023.09.007, PMID: 37673307

[10] Williams BA Law AD Routy B denHollander N Gupta V Wang XH. A phase I trial of NK-92 cells for refractory hematological Malignancies relapsing after autologous hematopoietic cell transplantation shows safety and evidence of efficacy. Oncotarget. (2017) 8:89256–68. doi: 10.18632/oncotarget.19204, PMID: 29179517 PMC5687687

[11] Goyco Vera D Waghela H Nuh M Pan J Lulla P. Approved CAR-T therapies have reproducible efficacy and safety in clinical practice. Hum Vaccin Immunother. (2024) 20:2378543. doi: 10.1080/21645515.2024.2378543, PMID: 39104200 PMC11305028

[12] Riess JW Lara MS Lopez de Rodas M Luxardi G Herbert S Shimoda M. Immune cell dynamics in EGFR-mutated NSCLC treated with afatinib and pembrolizumab: results from a phase IB study. JTO Clin Res Rep. (2024) 5:100706. doi: 10.1016/j.jtocrr.2024.100706, PMID: 39318388 PMC11420451

[13] Albinger N Hartmann J Ullrich E. Current status and perspective of CAR-T and CAR-NK cell therapy trials in Germany. Gene Ther. (2021) 28:513–27. doi: 10.1038/s41434-021-00246-w, PMID: 33753909 PMC8455322

[14] Heipertz EL Zynda ER Stav-Noraas TE Hungler AD Boucher SE Kaur N. Current perspectives on “Off-the-shelf” Allogeneic NK and CAR-NK cell therapies. Front Immunol. (2021) 12:732135. doi: 10.3389/fimmu.2021.732135, PMID: 34925314 PMC8671166

[15] Jhunjhunwala S Hammer C Delamarre L. Antigen presentation in cancer: insights into tumour immunogenicity and immune evasion. Nat Rev Cancer. (2021) 21:298–312. doi: 10.1038/s41568-021-00339-z, PMID: 33750922

[16] Herberman RB Holden HT Ting CC Lavrin DL Kirchner H. Cell-mediated immunity to leukemia virus- and tumor-associated antigens in mice. Cancer Res. (1976) 36:615–21. 56223

[17] Moretta L Montaldo E Vacca P Del Zotto G Moretta F Merli P. Human natural killer cells: origin, receptors, function, and clinical applications. Int Arch Allergy Immunol. (2014) 164:253–64. doi: 10.1159/000365632, PMID: 25323661

[18] Peng H Tian Z. NK cell trafficking in health and autoimmunity: a comprehensive review. Clin Rev Allergy Immunol. (2014) 47:119–27. doi: 10.1007/s12016-013-8400-0, PMID: 24366573

[19] Zhang Y Wallace DL de Lara CM Ghattas H Asquith B Worth A. *In vivo* kinetics of human natural killer cells: the effects of ageing and acute and chronic viral infection. Immunology. (2007) 121:258–65. doi: 10.1111/j.1365-2567.2007.02573.x, PMID: 17346281 PMC2265941

[20] Cherrier DE Serafini N Di Santo JP. Innate lymphoid cell development: A T cell perspective. Immunity. (2018) 48:1091–103. doi: 10.1016/j.immuni.2018.05.010, PMID: 29924975

[21] Wu SY Fu T Jiang YZ Shao ZM. Natural killer cells in cancer biology and therapy. Mol Cancer. (2020) 19:120. doi: 10.1186/s12943-020-01238-x, PMID: 32762681 PMC7409673

[22] Crinier A Narni-Mancinelli E Ugolini S Vivier E. SnapShot: natural killer cells. Cell. (2020) 180:1280–1280.e1281. doi: 10.1016/j.cell.2020.02.029, PMID: 32200803

[23] Dogra P Rancan C Ma W Toth M Senda T Carpenter DJ. Tissue determinants of human NK cell development, function, and residence. Cell. (2020) 180:749–763.e713. doi: 10.1016/j.cell.2020.01.022, PMID: 32059780 PMC7194029

[24] Siewiera J Gouilly J Hocine HR Cartron G Levy C Al-Daccak R. Natural cytotoxicity receptor splice variants orchestrate the distinct functions of human natural killer cell subtypes. Nat Commun. (2015) 6:10183. doi: 10.1038/ncomms10183, PMID: 26666685 PMC4682172

[25] Textor S Bossler F Henrich KO Gartlgruber M Pollmann J Fiegler N. The proto-oncogene Myc drives expression of the NK cell-activating NKp30 ligand B7-H6 in tumor cells. Oncoimmunology. (2016) 5:e1116674. doi: 10.1080/2162402x.2015.1116674, PMID: 27622013 PMC5007025

[26] Narni-Mancinelli E Gauthier L Baratin M Guia S Fenis A Deghmane AE. Complement factor P is a ligand for the natural killer cell-activating receptor NKp46. Sci Immunol. (2017) 2:eaam9628. doi: 10.1126/sciimmunol.aam9628, PMID: 28480349 PMC5419422

[27] Raskov H Orhan A Salanti A Gaggar S Gögenur I. Natural killer cells in cancer and cancer immunotherapy. Cancer Lett. (2021) 520:233–42. doi: 10.1016/j.canlet.2021.07.032, PMID: 34302920

[28] Bogovic Crncic T Laskarin G Juretic K Strbo N Dupor J Srsen S. Perforin and Fas/FasL cytolytic pathways at the maternal-fetal interface. Am J Reprod Immunol. (2005) 54:241–8. doi: 10.1111/j.1600-0897.2005.00320.x, PMID: 16212646

[29] Song B Aoki S Liu C Ito K. A toll-like receptor 9 agonist sensitizes mice to mitochondrial dysfunction-induced hepatic apoptosis via the Fas/FasL pathway. Arch Toxicol. (2019) 93:1573–84. doi: 10.1007/s00204-019-02454-1, PMID: 30993380

[30] Chin DS Lim CSY Nordin F Arifin N Jun TG. Antibody-dependent cell-mediated cytotoxicity through natural killer (NK) cells: unlocking NK cells for future immunotherapy. Curr Pharm Biotechnol. (2022) 23:552–78. doi: 10.2174/1389201022666210820093608, PMID: 34414871

[31] Abel AM Yang C Thakar MS Malarkannan S. Natural killer cells: development, maturation, and clinical utilization. Front Immunol. (2018) 9:1869. doi: 10.3389/fimmu.2018.01869, PMID: 30150991 PMC6099181

[32] Vivier E Raulet DH Moretta A Caligiuri MA Zitvogel L Lanier LL. Innate or adaptive immunity? The example of natural killer cells. Science. (2011) 331:44–9. doi: 10.1126/science.1198687, PMID: 21212348 PMC3089969

[33] Kelly JM Darcy PK Markby JL Godfrey DI Takeda K Yagita H. Induction of tumor-specific T cell memory by NK cell-mediated tumor rejection. Nat Immunol. (2002) 3:83–90. doi: 10.1038/ni746, PMID: 11743585

[34] Chu J Gao F Yan M Zhao S Yan Z Shi B. Natural killer cells: a promising immunotherapy for cancer. J Transl Med. (2022) 20:240. doi: 10.1186/s12967-022-03437-0, PMID: 35606854 PMC9125849

[35] Castriconi R Cantoni C Della Chiesa M Vitale M Marcenaro E Conte R. Transforming growth factor beta 1 inhibits expression of NKp30 and NKG2D receptors: consequences for the NK-mediated killing of dendritic cells. Proc Natl Acad Sci U.S.A. (2003) 100:4120–5. doi: 10.1073/pnas.0730640100, PMID: 12646700 PMC153058

[36] Ghiringhelli F Ménard C Terme M Flament C Taieb J Chaput N. CD4+CD25+ regulatory T cells inhibit natural killer cell functions in a transforming growth factor-beta-dependent manner. J Exp Med. (2005) 202:1075–85. doi: 10.1084/jem.20051511, PMID: 16230475 PMC2213209

[37] Laouar Y Sutterwala FS Gorelik L Flavell RA. Transforming growth factor-beta controls T helper type 1 cell development through regulation of natural killer cell interferon-gamma. Nat Immunol. (2005) 6:600–7. doi: 10.1038/ni1197, PMID: 15852008

[38] Landolina N Mariotti FR Pelosi A D’Oria V Ingegnere T Alicata C. The anti-inflammatory cytokine IL-37 improves the NK cell-mediated anti-tumor response. Oncoimmunology. (2024) 13:2297504. doi: 10.1080/2162402x.2023.2297504, PMID: 38170019 PMC10761114

[39] Xie G Dong H Liang Y Ham JD Rizwan R Chen J. CAR-NK cells: A promising cellular immunotherapy for cancer. EBioMedicine. (2020) 59:102975. doi: 10.1016/j.ebiom.2020.102975, PMID: 32853984 PMC7452675

[40] Wen J Chen Y Yang J Dai C Yu S Zhong W. Valproic acid increases CAR T cell cytotoxicity against acute myeloid leukemia. J Immunother Cancer. (2023) 11:e006857. doi: 10.1136/jitc-2023-006857, PMID: 37524506 PMC10391797

[41] Morris EC Neelapu SS Giavridis T Sadelain M. Cytokine release syndrome and associated neurotoxicity in cancer immunotherapy. Nat Rev Immunol. (2022) 22:85–96. doi: 10.1038/s41577-021-00547-6, PMID: 34002066 PMC8127450

[42] Pan K Farrukh H Chittepu V Xu H Pan CX Zhu Z. CAR race to cancer immunotherapy: from CAR T, CAR NK to CAR macrophage therapy. J Exp Clin Cancer Res. (2022) 41:119. doi: 10.1186/s13046-022-02327-z, PMID: 35361234 PMC8969382

[43] Marin D Li Y Basar R Rafei H Daher M Dou J. Safety, efficacy and determinants of response of allogeneic CD19-specific CAR-NK cells in CD19(+) B cell tumors: a phase 1/2 trial. Nat Med. (2024) 30:772–84. doi: 10.1038/s41591-023-02785-8, PMID: 38238616 PMC10957466

[44] Ramezani F Panahi Meymandi AR Akbari B Tamtaji OR Mirzaei H Brown CE. Outsmarting trogocytosis to boost CAR NK/T cell therapy. Mol Cancer. (2023) 22:183. doi: 10.1186/s12943-023-01894-9, PMID: 37974170 PMC10652537

[45] Zhang X Zhang C Qiao M Cheng C Tang N Lu S. Depletion of BATF in CAR-T cells enhances antitumor activity by inducing resistance against exhaustion and formation of central memory cells. Cancer Cell. (2022) 40:1407–1422.e1407. doi: 10.1016/j.ccell.2022.09.013, PMID: 36240777

[46] Yang S Sheffer M Kaplan IE Wang Z Tarannum M Dinh K. Non-pathogenic E. coli displaying decoy-resistant IL18 mutein boosts anti-tumor and CAR NK cell responses. Nat Biotechnol. (2024). doi: 10.1038/s41587-024-02418-6, PMID: 39367093 PMC12797303

[47] Egli L Kaulfuss M Mietz J Picozzi A Verhoeyen E Münz C. CAR T cells outperform CAR NK cells in CAR-mediated effector functions in head-to-head comparison. Exp Hematol Oncol. (2024) 13:51. doi: 10.1186/s40164-024-00522-6, PMID: 38745250 PMC11092129

[48] Wu W Ding S Mingming Z Yuping Z Sun X Zhao Z. Cost effectiveness analysis of CAR-T cell therapy for patients with relapsed/refractory multiple myeloma in China. J Med Econ. (2023) 26:701–9. doi: 10.1080/13696998.2023.2207742, PMID: 37145966

[49] Morabito F Martino EA Nizzoli ME Talami A Pozzi S Martino M. Comparative analysis of bispecific antibodies and CAR T-cell therapy in follicular lymphoma. Eur J Haematol. (2025) 114:4–16. doi: 10.1111/ejh.14335, PMID: 39462177 PMC11613673

[50] Guo C Chen H Yu J Lu H Xia Q Li X. Engagement of an optimized lentiviral vector enhances the expression and cytotoxicity of CAR in human NK cells. Mol Immunol. (2023) 155:91–9. doi: 10.1016/j.molimm.2023.01.010, PMID: 36736195

[51] Fujisaki H Kakuda H Shimasaki N Imai C Ma J Lockey T. Expansion of highly cytotoxic human natural killer cells for cancer cell therapy. Cancer Res. (2009) 69:4010–7. doi: 10.1158/0008-5472.Can-08-3712, PMID: 19383914 PMC2716664

[52] Denman CJ Senyukov VV Somanchi SS Phatarpekar PV Kopp LM Johnson JL. Membrane-bound IL-21 promotes sustained ex vivo proliferation of human natural killer cells. PloS One. (2012) 7:e30264. doi: 10.1371/journal.pone.0030264, PMID: 22279576 PMC3261192

[53] Baek HJ Kim JS Yoon M Lee JJ Shin MG Ryang DW. Ex vivo expansion of natural killer cells using cryopreserved irradiated feeder cells. Anticancer Res. (2013) 33:2011–9., PMID: 23645750

[54] Bae DS Lee JK. Development of NK cell expansion methods using feeder cells from human myelogenous leukemia cell line. Blood Res. (2014) 49:154–61. doi: 10.5045/br.2014.49.3.154, PMID: 25325034 PMC4188780

[55] Phan MT Lee SH Kim SK Cho D. Expansion of NK cells using genetically engineered K562 feeder cells. Methods Mol Biol. (2016) 1441:167–74. doi: 10.1007/978-1-4939-3684-7_14, PMID: 27177665

[56] Zhu H Kaufman DS. An improved method to produce clinical-scale natural killer cells from human pluripotent stem cells. Methods Mol Biol. (2019) 2048:107–19. doi: 10.1007/978-1-4939-9728-2_12, PMID: 31396935

[57] Thangaraj JL Phan MT Kweon S Kim J Lee JM Hwang I. Expansion of cytotoxic natural killer cells in multiple myeloma patients using K562 cells expressing OX40 ligand and membrane-bound IL-18 and IL-21. Cancer Immunol Immunother. (2022) 71:613–25. doi: 10.1007/s00262-021-02982-9, PMID: 34282497 PMC10991462

[58] Kang G Zhao X Sun J Cheng C Wang C Tao L. A2AR limits IL-15-induced generation of CD39+ NK cells with high cytotoxicity. Int Immunopharmacol. (2023) 114:109567. doi: 10.1016/j.intimp.2022.109567, PMID: 36529024

[59] Dolstra H Roeven MWH Spanholtz J Hangalapura BN Tordoir M Maas F. Successful transfer of umbilical cord blood CD34(+) hematopoietic stem and progenitor-derived NK cells in older acute myeloid leukemia patients. Clin Cancer Res. (2017) 23:4107–18. doi: 10.1158/1078-0432.Ccr-16-2981, PMID: 28280089

[60] Rutella S Muth J Vadakekulathu J Mathyer M Tumala B Foster M. 11P WU-NK-101: An enhanced NK cell therapy optimized for function in the tumor microenvironment (TME). Ann Oncol. (2022) 33:S549–50. doi: 10.1016/j.annonc.2022.07.039

[61] Multhoff G Seier S Stangl S Sievert W Shevtsov M Werner C. Targeted natural killer cell-based adoptive immunotherapy for the treatment of patients with NSCLC after radiochemotherapy: A randomized phase II clinical trial. Clin Cancer Res. (2020) 26:5368–79. doi: 10.1158/1078-0432.Ccr-20-1141, PMID: 32873573

[62] Fernandez RA Mayoral JE Pierre-Louis L Yao Y Xu Y Mu S. Improving NK cell function in multiple myeloma with NKTR-255, a novel polymer-conjugated human IL-15. Blood Adv. (2023) 7:9–19. doi: 10.1182/bloodadvances.2022007985, PMID: 35882498 PMC9813531

[63] Berrien-Elliott MM Becker-Hapak M Cashen AF Jacobs M Wong P Foster M. Systemic IL-15 promotes allogeneic cell rejection in patients treated with natural killer cell adoptive therapy. Blood. (2022) 139:1177–83. doi: 10.1182/blood.2021011532, PMID: 34797911 PMC9211446

[64] Granzin M Stojanovic A Miller M Childs R Huppert V Cerwenka A. Highly efficient IL-21 and feeder cell-driven ex vivo expansion of human NK cells with therapeutic activity in a xenograft mouse model of melanoma. Oncoimmunology. (2016) 5:e1219007. doi: 10.1080/2162402x.2016.1219007, PMID: 27757317 PMC5048763

[65] Nakazawa T Morimoto T Maeoka R Matsuda R Nakamura M Nishimura F. Establishment of an efficient ex vivo expansion strategy for human natural killer cells stimulated by defined cytokine cocktail and antibodies against natural killer cell activating receptors. Regener Ther. (2022) 21:185–91. doi: 10.1016/j.reth.2022.07.001, PMID: 35919498 PMC9309574

[66] Maude SL Laetsch TW Buechner J Rives S Boyer M Bittencourt H. Tisagenlecleucel in children and young adults with B-cell lymphoblastic leukemia. N Engl J Med. (2018) 378:439–48. doi: 10.1056/NEJMoa1709866, PMID: 29385370 PMC5996391

[67] Kalaitsidou M Kueberuwa G Schütt A Gilham DE. CAR T-cell therapy: toxicity and the relevance of preclinical models. Immunotherapy. (2015) 7:487–97. doi: 10.2217/imt.14.123, PMID: 26065475

[68] Qasim W. Allogeneic CAR T cell therapies for leukemia. Am J Hematol. (2019) 94:S50–s54. doi: 10.1002/ajh.25399, PMID: 30632623

[69] Lin C Zhang J. Reformation in chimeric antigen receptor based cancer immunotherapy: Redirecting natural killer cell. Biochim Biophys Acta Rev Cancer. (2018) 1869:200–15. doi: 10.1016/j.bbcan.2018.01.005, PMID: 29378229

[70] Lupo KB Matosevic S. Natural killer cells as allogeneic effectors in adoptive cancer immunotherapy. Cancers (Basel). (2019) 11:769. doi: 10.3390/cancers11060769, PMID: 31163679 PMC6628161

[71] Chou CK Turtle CJ. Assessment and management of cytokine release syndrome and neurotoxicity following CD19 CAR-T cell therapy. Expert Opin Biol Ther. (2020) 20:653–64. doi: 10.1080/14712598.2020.1729735, PMID: 32067497 PMC7393694

[72] Bhatnagar N Ahmad F Hong HS Eberhard J Lu IN Ballmaier M. FcγRIII (CD16)-mediated ADCC by NK cells is regulated by monocytes and FcγRII (CD32). Eur J Immunol. (2014) 44:3368–79. doi: 10.1002/eji.201444515, PMID: 25100508

[73] Morgan MA Büning H Sauer M Schambach A. Use of cell and genome modification technologies to generate improved “Off-the-shelf” CAR T and CAR NK cells. Front Immunol. (2020) 11:1965. doi: 10.3389/fimmu.2020.01965, PMID: 32903482 PMC7438733

[74] Jackson HJ Rafiq S Brentjens RJ. Driving CAR T-cells forward. Nat Rev Clin Oncol. (2016) 13:370–83. doi: 10.1038/nrclinonc.2016.36, PMID: 27000958 PMC5529102

[75] Hu Y Tian ZG Zhang C. Chimeric antigen receptor (CAR)-transduced natural killer cells in tumor immunotherapy. Acta Pharmacol Sin. (2018) 39:167–76. doi: 10.1038/aps.2017.125, PMID: 28880014 PMC5800464

[76] Pfefferle A Huntington ND. You have got a fast CAR: chimeric antigen receptor NK cells in cancer therapy. Cancers (Basel). (2020) 12:706. doi: 10.3390/cancers12030706, PMID: 32192067 PMC7140022

[77] Wang W Jiang J Wu C. CAR-NK for tumor immunotherapy: Clinical transformation and future prospects. Cancer Lett. (2020) 472:175–80. doi: 10.1016/j.canlet.2019.11.033, PMID: 31790761

[78] Li Y Hermanson DL Moriarity BS Kaufman DS. Human iPSC-derived natural killer cells engineered with chimeric antigen receptors enhance anti-tumor activity. Cell Stem Cell. (2018) 23:181–192.e185. doi: 10.1016/j.stem.2018.06.002, PMID: 30082067 PMC6084450

[79] Zenere G Olwenyi OA Byrareddy SN Braun SE. Optimizing intracellular signaling domains for CAR NK cells in HIV immunotherapy: a comprehensive review. Drug Discov Today. (2019) 24:983–91. doi: 10.1016/j.drudis.2019.02.002, PMID: 30771481 PMC7065919

[80] Buller CW Mathew PA Mathew SO. Roles of NK cell receptors 2B4 (CD244), CS1 (CD319), and LLT1 (CLEC2D) in cancer. Cancers (Basel). (2020) 12:1755. doi: 10.3390/cancers12071755, PMID: 32630303 PMC7409338

[81] Allison M Mathews J Gilliland T Mathew SO. Natural killer cell-mediated immunotherapy for leukemia. Cancers (Basel). (2022) 14:843. doi: 10.3390/cancers14030843, PMID: 35159109 PMC8833963

[82] Einsele H Schreder M. Treatment of multiple myeloma with the immunostimulatory SLAMF7 antibody elotuzumab. Ther Adv Hematol. (2016) 7:288–301. doi: 10.1177/2040620716657993, PMID: 27695618 PMC5026292

[83] Malaer JD Mathew PA. CS1 (SLAMF7, CD319) is an effective immunotherapeutic target for multiple myeloma. Am J Cancer Res. (2017) 7:1637–41., PMID: 28861320 PMC5574936

[84] Campbell KS Cohen AD Pazina T. Mechanisms of NK cell activation and clinical activity of the therapeutic SLAMF7 antibody, elotuzumab in multiple myeloma. Front Immunol. (2018) 9:2551. doi: 10.3389/fimmu.2018.02551, PMID: 30455698 PMC6230619

[85] Zhao Y Zhou X. Engineering chimeric antigen receptor-natural killer cells for cancer immunotherapy. Immunotherapy. (2020) 12:653–64. doi: 10.2217/imt-2019-0139, PMID: 32436428

[86] Wang X Yang X Yuan X Wang W Wang Y. Chimeric antigen receptor-engineered NK cells: new weapons of cancer immunotherapy with great potential. Exp Hematol Oncol. (2022) 11:85. doi: 10.1186/s40164-022-00341-7, PMID: 36324149 PMC9628181

[87] Imai C Iwamoto S Campana D. Genetic modification of primary natural killer cells overcomes inhibitory signals and induces specific killing of leukemic cells. Blood. (2005) 106:376–83. doi: 10.1182/blood-2004-12-4797, PMID: 15755898 PMC1895123

[88] Shimasaki N Fujisaki H Cho D Masselli M Lockey T Eldridge P. A clinically adaptable method to enhance the cytotoxicity of natural killer cells against B-cell Malignancies. Cytotherapy. (2012) 14:830–40. doi: 10.3109/14653249.2012.671519, PMID: 22458956

[89] Oelsner S Wagner J Friede ME Pfirrmann V Genßler S Rettinger E. Chimeric antigen receptor-engineered cytokine-induced killer cells overcome treatment resistance of pre-B-cell acute lymphoblastic leukemia and enhance survival. Int J Cancer. (2016) 139:1799–809. doi: 10.1002/ijc.30217, PMID: 27253354

[90] Suerth JD Morgan MA Kloess S Heckl D Neudörfl C Falk CS. Efficient generation of gene-modified human natural killer cells via alpharetroviral vectors. J Mol Med (Berl). (2016) 94:83–93. doi: 10.1007/s00109-015-1327-6, PMID: 26300042

[91] Klöß S Oberschmidt O Morgan M Dahlke J Arseniev L Huppert V. Optimization of human NK cell manufacturing: fully automated separation, improved ex vivo expansion using IL-21 with autologous feeder cells, and generation of anti-CD123-CAR-expressing effector cells. Hum Gene Ther. (2017) 28:897–913. doi: 10.1089/hum.2017.157, PMID: 28810809

[92] Oelsner S Friede ME Zhang C Wagner J Badura S Bader P. Continuously expanding CAR NK-92 cells display selective cytotoxicity against B-cell leukemia and lymphoma. Cytotherapy. (2017) 19:235–49. doi: 10.1016/j.jcyt.2016.10.009, PMID: 27887866

[93] Liu E Tong Y Dotti G Shaim H Savoldo B Mukherjee M. Cord blood NK cells engineered to express IL-15 and a CD19-targeted CAR show long-term persistence and potent antitumor activity. Leukemia. (2018) 32:520–31. doi: 10.1038/leu.2017.226, PMID: 28725044 PMC6063081

[94] Tang X Yang L Li Z Nalin AP Dai H Xu T. First-in-man clinical trial of CAR NK-92 cells: safety test of CD33-CAR NK-92 cells in patients with relapsed and refractory acute myeloid leukemia. Am J Cancer Res. (2018) 8:1083–9. PMC6048396 30034945

[95] Müller S Bexte T Gebel V Kalensee F Stolzenberg E Hartmann J. High cytotoxic efficiency of lentivirally and alpharetrovirally engineered CD19-specific chimeric antigen receptor natural killer cells against acute lymphoblastic leukemia. Front Immunol. (2019) 10:3123. doi: 10.3389/fimmu.2019.03123, PMID: 32117200 PMC7025537

[96] Jamali A Hadjati J Madjd Z Mirzaei HR Thalheimer FB Agarwal S. Highly efficient generation of transgenically augmented CAR NK cells overexpressing CXCR4. Front Immunol. (2020) 11:2028. doi: 10.3389/fimmu.2020.02028, PMID: 32983147 PMC7483584

[97] Liu Q Xu Y Mou J Tang K Fu X Li Y. Irradiated chimeric antigen receptor engineered NK-92MI cells show effective cytotoxicity against CD19(+) Malignancy in a mouse model. Cytotherapy. (2020) 22:552–62. doi: 10.1016/j.jcyt.2020.06.003, PMID: 32747298

[98] Liu E Marin D Banerjee P Macapinlac HA Thompson P Basar R. Use of CAR-transduced natural killer cells in CD19-positive lymphoid tumors. N Engl J Med. (2020) 382:545–53. doi: 10.1056/NEJMoa1910607, PMID: 32023374 PMC7101242

[99] Ghobadi A Bachanova V Patel K Park JH Flinn I Riedell PA. Induced pluripotent stem-cell-derived CD19-directed chimeric antigen receptor natural killer cells in B-cell lymphoma: a phase 1, first-in-human trial. Lancet. (2025) 405:127–36. doi: 10.1016/s0140-6736(24)02462-0, PMID: 39798981 PMC11827677

[100] Geller MA Cooley S Judson PL Ghebre R Carson LF Argenta PA. A phase II study of allogeneic natural killer cell therapy to treat patients with recurrent ovarian and breast cancer. Cytotherapy. (2011) 13:98–107. doi: 10.3109/14653249.2010.515582, PMID: 20849361 PMC3760671

[101] Yang Y Lim O Kim TM Ahn YO Choi H Chung H. Phase I study of random healthy donor-derived allogeneic natural killer cell therapy in patients with Malignant lymphoma or advanced solid tumors. Cancer Immunol Res. (2016) 4:215–24. doi: 10.1158/2326-6066.Cir-15-0118, PMID: 26787822

[102] Wrona E Borowiec M Potemski P. CAR-NK cells in the treatment of solid tumors. Int J Mol Sci. (2021) 22:5899. doi: 10.3390/ijms22115899, PMID: 34072732 PMC8197981

[103] Xiao L Cen D Gan H Sun Y Huang N Xiong H. Adoptive transfer of NKG2D CAR mRNA-engineered natural killer cells in colorectal cancer patients. Mol Ther. (2019) 27:1114–25. doi: 10.1016/j.ymthe.2019.03.011, PMID: 30962163 PMC6554529

[104] Velasquez MP Szoor A Bonifant CL Vaidya A Brunetti L Gundry MC. Two-pronged cell therapy for B-cell Malignancies: engineering NK cells to target CD22 and redirect bystander T cells to CD19. Blood. (2016 4560) 128:4560. doi: 10.1182/blood.v128.22.4560.4560

[105] Wang X Li J Wang Y Yang B Wei J Wu J. Efficient base editing in methylated regions with a human APOBEC3A-Cas9 fusion. Nat Biotechnol. (2018) 36:946–9. doi: 10.1038/nbt.4198, PMID: 30125268

[106] Bexte T Alzubi J Reindl LM Wendel P Schubert R Salzmann-Manrique E. CRISPR-Cas9 based gene editing of the immune checkpoint NKG2A enhances NK cell mediated cytotoxicity against multiple myeloma. Oncoimmunology. (2022) 11:2081415. doi: 10.1080/2162402x.2022.2081415, PMID: 35694192 PMC9176243

[107] Cao B Ni Q Chen Z Yang S Zhang X Su H. Development of glypican-3-specific chimeric antigen receptor-modified natural killer cells and optimization as a therapy for hepatocellular carcinoma. J Leukocyte Biol. (2024) 117:qiae144. doi: 10.1093/jleuko/qiae144, PMID: 38922297

[108] Liu Q Yang J Xing Y Zhao Y Liu Y. Development of delivery strategies for CRISPR-Cas9 genome editing. BMEMat. (2023) 1:e12025. doi: 10.1002/bmm2.12025

[109] Jain S Shukla S Yang C Zhang M Fatma Z Lingamaneni M. TALEN outperforms Cas9 in editing heterochromatin target sites. Nat Commun. (2021) 12:606. doi: 10.1038/s41467-020-20672-5, PMID: 33504770 PMC7840734

[110] Kerbauy LN Marin ND Kaplan M Banerjee PP Berrien-Elliott MM Becker-Hapak M. Combining AFM13, a bispecific CD30/CD16 antibody, with cytokine-activated blood and cord blood-derived NK cells facilitates CAR-like responses against CD30(+) Malignancies. Clin Cancer Res. (2021) 27:3744–56. doi: 10.1158/1078-0432.Ccr-21-0164, PMID: 33986022 PMC8254785

[111] Cichocki F Goodridge JP Bjordahl R Mahmood S Davis ZB Gaidarova S. Dual antigen-targeted off-the-shelf NK cells show durable response and prevent antigen escape in lymphoma and leukemia. Blood. (2022) 140:2451–62. doi: 10.1182/blood.2021015184, PMID: 35917442 PMC9918847

[112] Kim N Lee DH Choi WS Yi E Kim H Kim JM. Harnessing NK cells for cancer immunotherapy: immune checkpoint receptors and chimeric antigen receptors. BMB Rep. (2021) 54:44–58. doi: 10.5483/BMBRep.2021.54.1.214, PMID: 33298244 PMC7851441

[113] Lin M Luo H Liang S Chen J Liu A Niu L. Pembrolizumab plus allogeneic NK cells in advanced non-small cell lung cancer patients. J Clin Invest. (2020) 130:2560–9. doi: 10.1172/jci132712, PMID: 32027620 PMC7190908

[114] André P Denis C Soulas C Bourbon-Caillet C Lopez J Arnoux T. Anti-NKG2A mAb is a checkpoint inhibitor that promotes anti-tumor immunity by unleashing both T and NK cells. Cell. (2018) 175:1731–1743.e1713. doi: 10.1016/j.cell.2018.10.014, PMID: 30503213 PMC6292840

[115] Kamiya T Seow SV Wong D Robinson M Campana D. Blocking expression of inhibitory receptor NKG2A overcomes tumor resistance to NK cells. J Clin Invest. (2019) 129:2094–106. doi: 10.1172/jci123955, PMID: 30860984 PMC6486333

[116] Borst L van der Burg SH van Hall T. The NKG2A-HLA-E axis as a novel checkpoint in the tumor microenvironment. Clin Cancer Res. (2020) 26:5549–56. doi: 10.1158/1078-0432.Ccr-19-2095, PMID: 32409305

[117] Fayette J Bauman J Salas S Colevas D Even C Cupissol D. 81P Monalizumab in combination with cetuximab post platinum and anti-PD-(L) 1 in patients with recurrent/metastatic squamous cell carcinoma of the head and neck (R/M SCCHN): Updated results from a phase II trial. Ann Oncol. (2020) 31:S1450. doi: 10.1016/j.annonc.2020.10.568

[118] Romee R Rosario M Berrien-Elliott MM Wagner JA Jewell BA Schappe T. Cytokine-induced memory-like natural killer cells exhibit enhanced responses against myeloid leukemia. Sci Transl Med. (2016) 8:357ra123. doi: 10.1126/scitranslmed.aaf2341, PMID: 27655849 PMC5436500

[119] Lapteva N Durett AG Sun J Rollins LA Huye LL Fang J. Large-scale ex vivo expansion and characterization of natural killer cells for clinical applications. Cytotherapy. (2012) 14:1131–43. doi: 10.3109/14653249.2012.700767, PMID: 22900959 PMC4787300

[120] Myers JA Miller JS. Exploring the NK cell platform for cancer immunotherapy. Nat Rev Clin Oncol. (2021) 18:85–100. doi: 10.1038/s41571-020-0426-7, PMID: 32934330 PMC8316981

[121] Laskowski TJ Biederstädt A Rezvani K. Natural killer cells in antitumour adoptive cell immunotherapy. Nat Rev Cancer. (2022) 22:557–75. doi: 10.1038/s41568-022-00491-0, PMID: 35879429 PMC9309992

[122] Bari R Granzin M Tsang KS Roy A Krueger W Orentas R. A distinct subset of highly proliferative and lentiviral vector (LV)-transducible NK cells define a readily engineered subset for adoptive cellular therapy. Front Immunol. (2019) 10:2001. doi: 10.3389/fimmu.2019.02001, PMID: 31507603 PMC6713925

[123] Guven H Konstantinidis KV Alici E Aints A Abedi-Valugerdi M Christensson B. Efficient gene transfer into primary human natural killer cells by retroviral transduction. Exp Hematol. (2005) 33:1320–8. doi: 10.1016/j.exphem.2005.07.006, PMID: 16263416

[124] Streltsova MA Barsov E Erokhina SA Kovalenko EI. Retroviral gene transfer into primary human NK cells activated by IL-2 and K562 feeder cells expressing membrane-bound IL-21. J Immunol Methods. (2017) 450:90–4. doi: 10.1016/j.jim.2017.08.003, PMID: 28802832

[125] Quatrini L Mariotti FR Munari E Tumino N Vacca P Moretta L. The immune checkpoint PD-1 in natural killer cells: expression, function and targeting in tumour immunotherapy. Cancers (Basel). (2020) 12:3285. doi: 10.3390/cancers12113285, PMID: 33172030 PMC7694632

[126] Cózar B Greppi M Carpentier S Narni-Mancinelli E Chiossone L Vivier E. Tumor-infiltrating natural killer cells. Cancer Discov. (2021) 11:34–44. doi: 10.1158/2159-8290.Cd-20-0655, PMID: 33277307 PMC7611243

[127] Tong L Jiménez-Cortegana C Tay AHM Wickström S Galluzzi L Lundqvist A. NK cells and solid tumors: therapeutic potential and persisting obstacles. Mol Cancer. (2022) 21:206. doi: 10.1186/s12943-022-01672-z, PMID: 36319998 PMC9623927

[128] Galluzzi L Humeau J Buqué A Zitvogel L Kroemer G. Immunostimulation with chemotherapy in the era of immune checkpoint inhibitors. Nat Rev Clin Oncol. (2020) 17:725–41. doi: 10.1038/s41571-020-0413-z, PMID: 32760014

[129] Del Zotto G Marcenaro E Vacca P Sivori S Pende D Della Chiesa M. Markers and function of human NK cells in normal and pathological conditions. Cytometry B Clin Cytom. (2017) 92:100–14. doi: 10.1002/cyto.b.21508, PMID: 28054442

[130] Ng YY Du Z Zhang X Chng WJ Wang S. CXCR4 and anti-BCMA CAR co-modified natural killer cells suppress multiple myeloma progression in a xenograft mouse model. Cancer Gene Ther. (2022) 29:475–83. doi: 10.1038/s41417-021-00365-x, PMID: 34471234

[131] Chen C Wang Z Ding Y Qin Y. Tumor microenvironment-mediated immune evasion in hepatocellular carcinoma. Front Immunol. (2023) 14:1133308. doi: 10.3389/fimmu.2023.1133308, PMID: 36845131 PMC9950271

[132] Lei W Liu H Deng W Chen W Liang Y Gao W. Safety and feasibility of 4-1BB co-stimulated CD19-specific CAR-NK cell therapy in refractory/relapsed large B cell lymphoma: a phase 1 trial. Nat Cancer. (2025) 6:786–800. doi: 10.1038/s43018-025-00940-3, PMID: 40251398 PMC12122374

[133] Yao X Matosevic S. Cryopreservation of NK and T cells without DMSO for adoptive cell-based immunotherapy. BioDrugs. (2021) 35:529–45. doi: 10.1007/s40259-021-00494-7, PMID: 34427899 PMC12376086

[134] Wu X Matosevic S. Gene-edited and CAR-NK cells: Opportunities and challenges with engineering of NK cells for immunotherapy. Mol Ther Oncolytics. (2022) 27:224–38. doi: 10.1016/j.omto.2022.10.011, PMID: 36420307 PMC9676278

[135] Wang SW Gao C Zheng YM Yi L Lu JC Huang XY. Current applications and future perspective of CRISPR/Cas9 gene editing in cancer. Mol Cancer. (2022) 21:57. doi: 10.1186/s12943-022-01518-8, PMID: 35189910 PMC8862238

