# Supplementary material for: Recent advances in tumor immunotherapy based on NK cells
Source: Front Immunol. 2025 Aug 7;16:1595533. doi: 10.3389/fimmu.2025.1595533 (PMC12367655; doi:10.3389/fimmu.2025.1595533)
Supplement: Supplementary file 1 [file Table1.doc]

Supplementary Table 1. Clinical Studies on CAR-NK Cell Therapy in Hematologic Malignancies which have been registered on [www.clinicaltrials.gov](http://www.clinicaltrials.gov/).

| No. | NCT Number | Study Title | Study Status | Phases | Conditions | Interventions | City,  Country | Year | Web link |
| --- | --- | --- | --- | --- | --- | --- | --- | --- | --- |
| 1 | NCT05734898 | NKG2D CAR-NK & r/rAML | Unknown | NA | AML | Biological: NKG2D CAR-NK | Hangzhou, China | 2023 | <https://www.clinicaltrials.gov/study/>NCT05734898?cond=NCT05734898&rank=1 |
| 2 | NCT05941156 | Clinical Study of Anti-CD56-CAR-T in the Treatment of Relapsed/Refractory NK/T Cell Lymphoma /NK Cell Leukemia | Recruiting | Phase2 | Extranodal NK T Cell Lymphoma  NK-Cell Leukemia | Biological: Anti-CD56 CAR T | Xuzhou, China | 2023 | https://www.clinicaltrials.gov/study/NCT05941156?cond=NCT05941156&rank=1 |
| 3 | NCT06307054 | CLL-1 CAR-NK Cells for Relapsed/Refractory AML | Recruiting | Phase1 | Relapsed Adult AML  Refractory AML | Drug: anti-CLL-1 CAR NK cells | Shanghai, China | 2024 | https://www.clinicaltrials.gov/study/NCT06307054?cond=NCT06307054&rank=1 |
| 4 | NCT06690827 | Clinical Trial of CD123-targeted CAR-NK Therapy for Relapse/refractory AML or BPDCN | Recruiting | Phase1 | AML  BPDCN (blastic Plasmacytoid Dendritic Cell Neoplasm) | Drug: Anti-CD123 CAR NK cells | Hubei, China | 2024 | https://www.clinicaltrials.gov/study/NCT06690827?cond=NCT06690827&rank=1 |
| 5 | NCT05247957 | NKG2D CAR-NK Cell Therapy in Patients With Relapsed or Refractory Acute Myeloid Leukemia | Terminated | NA | Safety and Efficacy | Biological: CAR-NK cells | Hangzhou, China | 2021 | https://www.clinicaltrials.gov/study/NCT05247957?cond=NCT05247957&rank=1 |
| 6 | NCT06201247 | Off-the-shelf CD123 CAR-NK for R/R AML | Recruiting | Early Phase1 | AML, in Relapse  AML Refractory | Drug: JD123 injection | Beijing, China | 2023 | https://www.clinicaltrials.gov/study/NCT06201247?cond=NCT06201247&rank=1 |
| 7 | NCT05570188 | Anti-CD19 Universal CAR-NK Cells Therapy Combined With HSCT for B Cell Hematologic Malignancies | Withdrawn | Phase1  Phase2 | B-cell Lymphoma  B-cell Leukemia | Biological: anti-CD19 UCAR-NK cells | Kunming, China | 2022 | https://www.clinicaltrials.gov/study/NCT05570188?cond=NCT05570188&rank=1 |
| 8 | NCT06631040 | Cell Therapy with Anti-CD19 CAR-NK Cells in Patients with Relapsed or Resistant B-ALL | Not Yet Recruiting | Phase1  Phase2 | AML in Relapse | Biological: anti CD19 CAR NK cells | Iran, Islamic Republic | 2024 | https://www.clinicaltrials.gov/study/NCT06631040?cond=NCT06631040&rank=1#contacts-and-locations |
| 9 | NCT05645601 | CAR-NK Targeted CD19 for r/r B-cell Malignancies | Unknown | Phase1 | Adult Relapsed/Refractory B-cell Hematologic Malignancies | Biological: CD19-CAR-NK | Beijing, China | 2022 | https://www.clinicaltrials.gov/study/NCT05645601?cond=NCT05645601&rank=1 |
| 10 | NCT06827782 | Cord Blood-derived CAR-NK Cells Targeting CD19 for Refractory/relapsed Central Nervous System Lymphoma | Enrolling By Invitation | Phase1 | Refractory/recurrent Central Nervous System Lymphoma | Biological: anti-CD19 CAR-NK cells | Hangzhou, China | 2025 | https://www.clinicaltrials.gov/study/NCT06827782?cond=NCT06827782&rank=1 |
| 11 | NCT05654038 | A Study of Universal CD19-Targeted UCAR-NK Cells Combined With HSCT for B Cell Hematologic Malignancies | Unknown | Phase1  Phase2 | B-Cell Lymphoblastic Leukemia/Lymphoma | Biological: Anti-CD19 UCAR-NK cells | Kunming, China | 2022 | https://www.clinicaltrials.gov/study/NCT05654038?cond=NCT05654038&rank=1 |
| 12 | NCT05215015 | Study of Anti-CD33/CLL1 CAR-NK in Acute Myeloid Leukemia | Unknown | Early Phase1 | Acute Myeloid Leukemia | Biological: Anti-CD33/CLL1 CAR-NK Cells | Wuxi, China | 2020 | https://www.clinicaltrials.gov/study/NCT05215015?cond=NCT05215015&rank=1 |
| 13 | NCT06696846 | CD70-CAR-NK Cell Therapy for T Cell Lymphoma and Acute Myeloid Leukemia | Not Yet Recruiting | Phase1 | Relapsed/refractory T-cell Lymphoma|Relapsed/refractory Acute Myeloid Leukemia | Biological: CD70 CAR-NK | Hangzhou, China | 2024 | https://www.clinicaltrials.gov/study/NCT06696846?cond=NCT06696846&rank=1 |
| 14 | NCT05842707 | Study of Cord Blood-derived CAR NK Cells Targeting CD19/CD70 in Refractory/Relapsed B-cell Non-Hodgkin Lymphoma | Recruiting | Phase1  Phase2 | Refractory or Relapsed B-cell Non-Hodgkin Lymphoma | Drug: dualCAR-NK19/70 cell | Shanghai, China | 2023 | https://www.clinicaltrials.gov/study/NCT05842707?cond=NCT05842707&rank=1 |
| 15 | NCT04639739 | Anti-CD19 CAR NK Cell Therapy for R/R Non-Hodgkin Lymphoma. | Unknown | Early Phase1 | NHL | Biological: anti-CD19 CAR NK | Chongqing, China | 2020 | https://www.clinicaltrials.gov/study/NCT04639739?cond=NCT04639739&rank=1 |
| 16 | NCT05410041 | Anti-CD19 CAR-Engineered NK Cells in the Treatment of Relapsed/Refractory B-cell Malignancies | Unknown | Phase1 | Acute Lymphocytic Leukemia  Chronic Lymphocytic Leukemia  Non Hodgkin Lymphoma | Biological: CAR-NK-CD19 Cells | Beijing, China | 2022 | https://www.clinicaltrials.gov/study/NCT05410041?cond=NCT05410041&rank=1 |
| 17 | NCT05008575 | Anti-CD33 CAR NK Cells in the Treatment of Relapsed/Refractory Acute Myeloid Leukemia | Unknown | Phase1 | Leukemia, Myeloid, Acute | Biological: anti-CD33 CAR NK cells  Drug: Fludarabine  Drug: Cytoxan | Chongqing, China | 2021 | https://www.clinicaltrials.gov/study/NCT05008575?cond=NCT05008575&rank=1 |
| 18 | NCT04004637 | CD7 CAR-T Cells for Patients With R/R CD7+ NK/T Cell Lymphoma,T-lymphoblastic Lymphoma and Acute Lymphocytic Leukemia | Unknown | Phase1 | T-lymphoblastic Lymphoma  NK/T Cell Lymphoma  Acute Lymphocytic Leukemia | Drug: CD7 CAR-T cells infusion | Suzhou, China | 2019 | https://www.clinicaltrials.gov/study/NCT04004637?cond=NCT04004637&rank=1 |
| 19 | NCT05574608 | Allogenic CD123-CAR-NK Cells in the Treatment of Refractory/Relapsed Acute Myeloid Leukemia | Recruiting | Early Phase1 | AML Refractory  AML Recurrent | Biological: CD123-CAR-NK cells | Beijing, China | 2022 | https://www.clinicaltrials.gov/study/NCT05574608?cond=NCT05574608&rank=1 |
| 20 | NCT05673447 | The Study of Anti-CD19 CAR NK Cells in the Treatment of Relapsed/Refractory Diffuse Large B Cell Lymphoma | Unknown | Early Phase1 | Diffuse Large B Cell Lymphoma | Biological: anti-CD19 CAR NK cells | Shanghai, China | 2023 | https://www.clinicaltrials.gov/study/NCT05673447?cond=NCT05673447&rank=1 |
| 21 | NCT05739227 | Safety and Efficacy of Allogenic CD19-CAR-NK Cells in Treatmenting r/r B-cell Hematologic Malignancies | Recruiting | Early Phase1 | Acute Lymphoblastic Leukemia  B-cell Lymphoma  Chronic Lymphocytic Leukemia | Other: allogenic CD19-CAR-NK cells | Xuzhou, China | 2023 | https://www.clinicaltrials.gov/study/NCT05739227?cond=NCT05739227&rank=1 |
| 22 | NCT06707259 | Clinical Study of Cord Blood-derived IL-10 CD19-CAR NK in the Treatment of Refractory/Relapsed B-cell NHL | Recruiting | Phase1 | B-cell Non Hodgkin Lymphoma | Biological: anti-CD19 IL10 CAR-NK | Hangzhou, China | 2024 | https://www.clinicaltrials.gov/study/NCT06707259?cond=NCT06707259&rank=1 |
| 23 | NCT04264078 | Anti-CD7 U-CAR-T Cell Therapy for T/NK Cell Hematologic Malignancies | Unknown | Early Phase1 | T-cell Leukemia  T-cell Lymphoma | Biological: CD7 UCAR-T cells  Drug: Fludarabine  Drug: Cytoxan  Drug: Melphalan | Chongqing, China | 2021 | https://www.clinicaltrials.gov/study/NCT04264078?cond=NCT04264078&rank=1 |
| 24 | NCT04033302 | Multi-CAR T Cell Therapy Targeting CD7-positive Malignancies | Unknown | Phase1  Phase2 | T-cell Acute Lymphoblastic Leukemia  T-cell Acute Lymphoblastic Lymphoma  AML  NK Cell Lymphoma | Biological: CD7-specific CAR gene-engineered T cells | Shenzhen, China | 2019 | https://www.clinicaltrials.gov/study/NCT04033302?cond=NCT04033302&rank=1 |
| 25 | NCT03692767 | Study of Anti-CD22 CAR NK Cells in Relapsed and Refractory B Cell Lymphoma | Unknown | Early Phase1 | Refractory B-Cell Lymphoma | Biological: Anti-CD22 CAR NK Cells | Beijing, China | 2019 | https://www.clinicaltrials.gov/study/NCT03692767?cond=NCT03692767&rank=1 |
| 26 | NCT03690310 | Study of Anti-CD19 CAR NK Cells in Relapsed and Refractory B Cell Lymphoma | Unknown | Early Phase1 | Refractory B-Cell Lymphoma | Biological: Anti-CD19 CAR NK Cells | Beijing, China | 2019 | https://www.clinicaltrials.gov/study/NCT03690310?cond=NCT03690310&rank=1 |
| 27 | NCT05008536 | Anti-BCMA CAR-NK Cell Therapy for the Relapsed or Refractory Multiple Myeloma | Unknown | Early Phase1 | Multiple Myeloma, Refractory | Biological: Anti-BCMA CAR-NK Cells  Drug: Fludarabine  Drug: Cytoxan | Chongqing, China | 2021 | https://www.clinicaltrials.gov/study/NCT05008536?cond=NCT05008536&rank=1 |
| 28 | NCT04887012 | Clinical Study of HLA Haploidentical CAR-NK Cells Targeting CD19 in the Treatment of Refractory/Relapsed B-cell NHL | Unknown | Phase1 | B-cell Non Hodgkin Lymphoma | Biological: anti-CD19 CAR-NK | Hangzhou, China | 2021 | https://www.clinicaltrials.gov/study/NCT04887012?cond=NCT04887012&rank=1 |
| 29 | NCT05667155 | Clinical Study of Cord Blood-derived CAR NK Cells Targeting CD19/CD70 in Refractory/Relapsed B-cell Non-Hodgkin Lymphoma | Recruiting | Phase1 | B-cell Non Hodgkin Lymphoma | Biological: CB dual CAR-NK19/70 | Hangzhou, China | 2022 | https://www.clinicaltrials.gov/study/NCT05667155?cond=NCT05667155&rank=1 |
| 30 | NCT05472558 | Clinical Study of Cord Blood-derived CAR-NK Cells Targeting CD19 in the Treatment of Refractory/Relapsed B-cell NHL | Recruiting | Phase1 | B-cell Non Hodgkin Lymphoma | Biological: anti-CD19 CAR-NK | Hangzhou, China | 2022 | https://www.clinicaltrials.gov/study/NCT05472558?cond=NCT05472558&rank=1 |
| 31 | NCT04623944 | NKX101, Intravenous Allogeneic CAR NK Cells, in Adults With AML or MDS | Active Not Recruiting | Phase1 | Relapsed/Refractory AML  AML, Adult  MDS  Refractory Myelodysplastic Syndromes | Biological: NKX101 - CAR NK cell therapy | Colorado, USA | 2020 | https://www.clinicaltrials.gov/study/NCT04623944?cond=NCT04623944&rank=1 |
| 32 | NCT03824964 | Study of Anti-CD19/CD22 CAR NK Cells in Relapsed and Refractory B Cell Lymphoma | Unknown | Early Phase1 | Refractory B-Cell Lymphoma | Biological: Anti-CD19/CD22 CAR NK Cells | Beijing, China | 2019 | https://www.clinicaltrials.gov/study/NCT03824964?cond=NCT03824964&rank=1 |
| 33 | NCT06325748 | SENTI-202: Off-the-shelf Logic Gated CAR NK Cell Therapy in Adults With CD33 and/or FLT3 Blood Cancers Including AML/MDS | Recruiting | Phase1 | AML/MDS  CD33 Expressing Hematological Malignancies  FLT3 Expressing Hematological Malignancies | Biological: SENTI-202 | California, USA | 2024 | https://www.clinicaltrials.gov/study/NCT06325748?cond=NCT06325748&rank=1 |
| 34 | NCT06594211 | Allogeneic Anti-BCMA/GPRC5D Bispecific CAR-NK Cells (ACT-001) in Patients With Relapsed or Refractorymultiple Myeloma | Not Yet Recruiting | NA | Multiple Myeloma | Biological: ACT-001 CAR-NK cell | Shanghai, China | 2024 | https://www.clinicaltrials.gov/study/NCT06594211?cond=NCT06594211&rank=1 |
| 35 | NCT06045091 | To Evaluate the Safety and Efficacy of Human BCMA Targeted CAR-NK Cells Injection for Subjects With R/R MM or PCL | Recruiting | Early Phase1 | Multiple Myeloma  Plasma Cell Leukemia | Drug: Human BCMA targeted CAR-NK cells injection | Shanghai, China | 2023 | https://www.clinicaltrials.gov/study/NCT06045091?cond=NCT06045091&rank=1 |
| 36 | NCT05110742 | Phase I/II Study of CD5 CAR Engineered IL15-Transduced Cord Blood-Derived NK Cells in Conjunction With Lymphodepleting Chemotherapy for the Management of Relapsed/Refractory Hematological Malignances | Recruiting | Phase1  Phase2 | Hematological Malignancy | Drug: Fludarabine Phosphate  Drug: Cyclophosphamide  Drug: CAR.5/IL15-transduced CB-NK cells | Houston, USA | 2024 | https://www.clinicaltrials.gov/study/NCT05110742?cond=NCT05110742&rank=1 |
| 37 | NCT03056339 | Umbilical & Cord Blood (CB) Derived CAR-Engineered NK Cells for B Lymphoid Malignancies | Completed | Phase1  Phase2 | B-Lymphoid Malignancies|Acute Lymphocytic Leukemia|Chronic Lymphocytic Leukemia|Non-hodgkin Lymphoma | Drug: Fludarabine  Drug: Cyclophosphamide  Drug: Mesna  Biological: iC9/CAR.19/IL15-Transduced CB-NK Cells  Drug: AP1903 | Houston, USA | 2017 | https://www.clinicaltrials.gov/study/NCT03056339?cond=NCT03056339&rank=1 |
| 38 | NCT04796675 | Cord Blood Derived Anti-CD19 CAR-Engineered NK Cells for B Lymphoid Malignancies | Unknown | Phase1 | Acute Lymphocytic Leukemia  Chronic Lymphocytic Leukemia  Non Hodgkin's Lymphoma | Drug: Fludarabine + Cyclophosphamide + CAR-NK-CD19 Cells | Hubei, China | 2021 | https://www.clinicaltrials.gov/study/NCT04796675?cond=NCT04796675&rank=1 |
| 39 | NCT06006403 | Safety and Efficacy of CD123-targeted CAR-NK for Relapsed/Refractory Acute Myeloid Leukemia or Blastic Plasmacytoid Dendritic Cell Neoplasm | Recruiting | Phase1  Phase2 | Acute Myeloid Leukemia  Blastic Plasmacytoid Dendritic Cell Neoplasm (BPDCN)  Relapse Leukemia|Refractory Leukemia | Biological: CD123 targeted CAR-NK cells | Chongqing, China | 2023 | https://www.clinicaltrials.gov/study/NCT06006403?cond=NCT06006403&rank=1 |
| 40 | NCT05563545 | Anti-CD19 CAR-Engineered NK Cells in the Treatment of Relapsed/Refractory Acute Lymphoblastic Leukemia | Completed | Phase1 | Acute Lymphoblastic Leukemia | Biological: CAR-NK-CD19 Cells | Shanghai, China | 2022 | https://www.clinicaltrials.gov/study/NCT05563545?cond=NCT05563545&rank=1 |
| 41 | NCT06379451 | An Clinical Study of NKG2D-CAR-NK Cells for the Treatment of Refractory Recurrent Multiple Myeloma | Not Yet Recruiting | Early Phase1 | Multiple Myeloma | Drug: NKG2D Chimeric Antigen Receptor NK Cell Injection | Changzhou, China | 2024 | https://www.clinicaltrials.gov/study/NCT06379451?cond=NCT06379451&rank=1 |
| 42 | NCT05020678 | NKX019, Intravenous Allogeneic Chimeric Antigen Receptor Natural Killer Cells (CAR NK), in Adults With B-cell Cancers | Active Not Recruiting | Phase1 | Lymphoma, Non-Hodgkin  B-cell Acute Lymphoblastic Leukemia  Large B-cell Lymphoma  Mantle Cell Lymphoma  Indolent Lymphoma  Waldenstrom Macroglobulinemia  Chronic Lymphocytic Leukemia  Small Lymphocytic Lymphoma  Aggressive Lymphoma  Large-cell Lymphoma | Biological: NKX019 | Colorado, USA | 2021 | https://www.clinicaltrials.gov/study/NCT05020678?cond=NCT05020678&rank=1 |
| 43 | NCT02944162 | CAR-pNK Cell Immunotherapy for Relapsed/Refractory CD33+ AML | Unknown | Phase1  Phase2 | Acute Myelogenous Leukemia  Acute Myeloid Leukemia  Acute Myeloid Leukemia With Maturation  Acute Myeloid Leukemia Without Maturation  ANLL | Biological: anti-CD33 CAR-NK cells | Suzhou, China | 2016 | https://www.clinicaltrials.gov/study/NCT02944162?cond=NCT02944162&rank=1 |
| 44 | NCT02274584 | CAR T Cells Targeting CD30 Positive Lymphomas (4SCAR30273) | Unknown | Phase1  Phase2 | Lymphomas | Genetic: Anti-CD30 CAR T cells | Beijing, China | 2014 | https://www.clinicaltrials.gov/study/NCT02274584?cond=NCT02274584&rank=1 |
| 45 | NCT04747093 | Induced-T Cell Like NK Cells for B Cell Malignancies | Unknown | Phase1  Phase2 | B Cell Leukemia  B Cell Lymphoma  B-cell Acute Lymphoblastic Leukemia  B-cell Lymphoma Recurrent  B-cell Lymphoma Refractory | Biological: CAR-ITNK cells | Guangzhou, China | 2021 | https://www.clinicaltrials.gov/study/NCT04747093?cond=NCT04747093&rank=1 |
| 46 | NCT05995028 | Universal 4SCAR7U Targeting CD7-positive Malignancies | Recruiting | Phase1 | T-cell Acute Lymphoblastic Leukemia  T-cell Acute Lymphoblastic Lymphoma  Acute Myeloid Leukemia  NK Cell Lymphoma | Biological: Universal CD7-specific CAR gene-engineered T cells | Shenzhen, China | 2023 | https://www.clinicaltrials.gov/study/NCT05995028?cond=NCT05995028&rank=1 |
| 47 | NCT04526834 | Phase 1 Study of Autologous CD30.CAR-T in Relapsed or Refractory CD30 Positive Non-Hodgkin Lymphoma | Active Not Recruiting | Phase1 | Anaplastic Large Cell Lymphoma  Peripheral T Cell Lymphoma  Extranodal NK/T-cell Lymphoma  Diffuse Large B Cell Lymphoma  Primary Mediastinal Large B-Cell Lymphoma (PMBCL) | Drug: CD30.CAR-T | California, USA | 2021 | https://www.clinicaltrials.gov/study/NCT04526834?cond=NCT04526834&rank=1 |
| 48 | NCT05208853 | An Exploratory Clinical Study Evaluating the Safety and Efficacy of Anti CD30 CAR T Cells in Patients With CD30+ Relapsed/Refractory Lymphoma | Unknown | Early Phase1 | Hodgkin Lymphoma  NK/T Cell Lymphoma  Peripheral T Cell Lymphoma, Unspecified  Angioimmunoblastic T-cell Lymphoma  Anaplastic Large Cell Lymphoma  Diffuse Large B Cell Lymphoma  Mediastinal B-Cell Diffuse Large Cell Lymphoma  Gray Zone Lymphoma | Biological: Anti CD30 CAR-T Cell Injection | Hangzhou, China | 2022 | https://www.clinicaltrials.gov/study/NCT05208853?cond=NCT05208853&rank=1 |
| 49 | NCT04008394 | Anti-CD30 CAR-T Therapy in Patients With Refractory/Relapsed Lymphocyte Malignancies | Unknown | Phase1 | Adult T-Cell Lymphoma/Leukaemia  Anaplastic Large Cell Lymphoma  Angioimmunoblastic T-cell Lymphoma  NK/T-cell Lymphoma  Peripheral T Cell Lymphoma  Hodgkin Lymphoma | Genetic: Anti-CD30 CAR T cells | Wuhan, China | 2019 | https://www.clinicaltrials.gov/study/NCT04008394?cond=NCT04008394&rank=1 |
| 50 | NCT03579927 | CAR.CD19-CD28-zeta-2A-iCasp9-IL15-Transduced Cord Blood NK Cells, High-Dose Chemotherapy, and Stem Cell Transplant in Treating Participants With B-cell Lymphoma | Withdrawn | Phase1  Phase2 | CD19 Positive  Mantle Cell Lymphoma  Recurrent Diffuse Large B-Cell Lymphoma  Recurrent Follicular Lymphoma  Refractory B-Cell Non-Hodgkin Lymphoma  Refractory Diffuse Large B-Cell Lymphoma  Refractory Follicular Lymphoma | Procedure: Autologous Hematopoietic Stem Cell Transplantation  Drug: Carmustine  Drug: Cytarabine  Drug: Etoposide  Biological: Filgrastim  Drug: Melphalan  Biological: Rituximab  Biological: Umbilical Cord Blood-derived Natural Killer Cells | Houston, USA | 2019 | https://www.clinicaltrials.gov/study/NCT03579927?cond=NCT03579927&rank=1 |
| 51 | NCT03049449 | T Cells Expressing a Fully-Human Anti-CD30 Chimeric Antigen Receptor for Treating CD30-Expressing Lymphomas | Completed | Phase1 | Lymphoma, Large-Cell, Anaplastic  Enteropathy-Associated T-Cell Lymphoma  Lymphoma, Large B-Cell, Diffuse  Lymphoma, Extranodal NK-T-Cell  Lymphoma, T-Cell, Peripheral | Biological: Anti-Tumor Necrosis Factor (TNF) Receptor Superfamily Member 8 (CD30) Chimeric Antigen Receptor (CAR) T cells  Drug: Cyclophosphamide  Drug: Fludarabine | Bethesda, USA | 2017 | https://www.clinicaltrials.gov/study/NCT03049449?cond=NCT03049449&rank=1 |
| 52 | NCT05487651 | Allogeneic NK T-Cells Expressing CD19 Specific CAR in B-Cell Malignancies | Recruiting | Phase1 | NHL, Relapsed, Adult  B-cell Lymphoma  B-cell Leukemia  DLBCL - Diffuse Large B Cell Lymphoma  ALL, Adult B Cell  ALL, Childhood  CLL/SLL | Genetic: KUR-502 | California, USA | 2022 | https://www.clinicaltrials.gov/study/NCT05487651?cond=NCT05487651&rank=1 |
| 53 | NCT05092451 | Phase I/II Study of CAR.70- Engineered IL15-transduced Cord Blood-derived NK Cells in Conjunction With Lymphodepleting Chemotherapy for the Management of Relapse/Refractory Hematological Malignances | Recruiting | Phase1  Phase2 | B-Cell Lymphoma  Myelodysplastic Syndromes (MDS)  Acute Myeloid Leukemia (AML)  Multiple Myeloma  Plasma Cell Leukemia  Hodgkin Lymphoma  T-cell Non-Hodgkin's Lymphoma/ T-cell Acute Lymphoblastic Leukmeia  Myelodysplastic Syndrome / Chronic Myelomonocytic Leukemia  Blastic Transformation of Chronic Myeloid Leukemia  Germ Cell Tumors | Drug: Cyclophosphamide  Drug: CAR.70/IL15-transduced CB-NK cells  Drug: Fludarabine phosphate | Houston, USA | 2022 | https://www.clinicaltrials.gov/study/NCT05092451?cond=NCT05092451&rank=1 |
| 54 | NCT02742727 | CAR-pNK Cell Immunotherapy in CD7 Positive Leukemia and Lymphoma | Unknown | Phase1  Phase2 | Acute Myeloid Leukemia  Precursor T-Cell Lymphoblastic Leukemia-Lymphoma  T-cell Prolymphocytic Leukemia  T-cell Large Granular Lymphocytic Leukemia  Peripheral T-cell Lymphoma, NOS  Angioimmunoblastic T-cell Lymphoma  Extranodal NK/T-cell Lymphoma, Nasal Type  Enteropathy-type Intestinal T-cell Lymphoma  Hepatosplenic T-cell Lymphoma | Biological: anti-CD7 CAR-pNK cells | Suzhou, China | 2016 | https://www.clinicaltrials.gov/study/NCT02742727?cond=NCT02742727&rank=1 |
| 55 | NCT02134262 | Gene Therapy for B-Cell Non-Hodgkin Lymphoma Using CD19 CAR Gene Transduced T Lymphocytes | Unknown | Phase1  Phase2 | Relapsed or Refractory B-Cell Non-Hodgkin Lymphoma | Drug: Cyclophosphamide or Bendamustine  Genetic: Dose Level -1  Genetic: Dose Level 1  Genetic: Dose Level 2  Genetic: Dose Level 3 | Tochigi, Japan | 2014 | https://www.clinicaltrials.gov/study/NCT02134262?cond=NCT02134262&rank=1 |
| 56 | NCT05336409 | A Study of CNTY-101 in Participants With CD19-Positive B-Cell Malignancies | Recruiting | Phase1 | R/R CD19-Positive B-Cell Malignancies  Indolent Non-Hodgkin Lymphoma  Aggressive Non-Hodgkin Lymphoma | Biological: CNTY-101  Biological: IL-2  Drug: Lymphodepleting Chemotherapy | Arizona, USA | 2023 | https://www.clinicaltrials.gov/study/NCT05336409?cond=NCT05336409&rank=1 |
| 57 | NCT05377827 | Dose-Escalation and Dose-Expansion Study to Evaluate the Safety and Tolerability of Anti-CD7 Allogeneic CAR T-Cells (WU-CART-007) in Patients With CD7+ Hematologic Malignancies | Recruiting | Phase1 | T-Cell Non-Hodgkin Lymphoma  Acute Myeloid Leukemia  Angioimmunoblastic T-cell Lymphoma  Enteropathy-Associated T-Cell Lymphoma  Monomorphic Epitheliotropic Intestinal T-Cell Lymphoma  Peripheral T Cell Lymphoma  Anaplastic Large Cell Lymphoma  Adult T Cell Leukemia  Adult T Cell Lymphoma  T Cell Prolymphocytic Leukemia  Extranodal NK/T-cell Lymphoma  Transformed Mycosis Fungoides  Sezary Syndrome  Primary Cutaneous Gamma-Delta T-Cell Lymphoma  Hepatosplenic T-cell Lymphoma | Biological: WU-CART-007 | Saint Louis, USA | 2023 | https://www.clinicaltrials.gov/study/NCT05377827?cond=NCT05377827&rank=1 |
| 58 | NCT04480788 | CD7-CART in the Treatment of r / r CD7 Positive Hemolymph System Malignancies on Increasing Dose and Open Label Study | Unknown | Phase1 | T Lymphoblastic Leukemia/Lymphoma  Extramedullary NK-T-cell Lymphoma, Nasal Type  Peripheral T-cell Lymphoma, Nonspecific  Angioimmunoblastic T-cell Lymphoma  Enteropathy-Associated T-Cell Lymphoma  Anaplastic Large Cell Lymphoma, ALK-negative  T-cell Lymphoblastic Leukemia | Biological: T cell injection targeting CD7 chimeric antigen receptor | Suzhou, China | 2020 | https://www.clinicaltrials.gov/study/NCT04480788?cond=NCT04480788&rank=1 |
